# A Specialized Microvascular Domain in the Mouse Neural Stem Cell Niche

**DOI:** 10.1371/journal.pone.0053546

**Published:** 2013-01-07

**Authors:** James C. Culver, Tegy J. Vadakkan, Mary E. Dickinson

**Affiliations:** Department of Molecular Physiology and Biophysics, Baylor College of Medicine, Houston, Texas, United States of America; Institut National de la Recherche Agronomique-CNRS UMR6175, France

## Abstract

The microenvironment of the subependymal zone (SEZ) neural stem cell niche is necessary for regulating adult neurogenesis. In particular, signaling from the microvasculature is essential for adult neural stem cell maintenance, but microvascular structure and blood flow dynamics in the SEZ are not well understood. In this work, we show that the mouse SEZ constitutes a specialized microvascular domain defined by unique vessel architecture and reduced rates of blood flow. Additionally, we demonstrate that hypoxic conditions are detectable in the ependymal layer that lines the ventricle, and in a subpopulation of neurons throughout the SEZ and striatum. Together, these data highlight previously unidentified features of the SEZ neural stem cell niche, and further demonstrate the extent of microvascular specialization in the SEZ microenvironment.

## Introduction

Adult neurogenesis in mammals is mostly restricted to two specialized regions of the brain: the subependymal zone (SEZ), which is located just beneath the ependymal wall of the lateral ventricles, and the subgranular zone (SGZ) of the hippocampus [1]–[4]. The SEZ comprises the largest population of neural stem cells (NSCs) in the adult brain, and is derived from a subset of the NSCs that populate the cortical layers of the developing embryonic and postnatal brain [2], [5], [6]. Under physiological conditions, neuroblasts produced in this region are incorporated into the olfactory bulb in mice, or into the prefrontal cortex in human infants [2], [7], [8]. Experiments using rodent models of stroke, however, have shown that these neuroblasts can also integrate with brain tissue at sites of damage [9], [10], suggesting that the SEZ may have some limited capacity for brain repair. One principle that has emerged from studies of the SEZ is that the microenvironment is a crucial regulator of neurogenesis [6], [11]–[15]. While several components of the SEZ microenvironment contribute to this function, including a variety of cell types, extracellular matrix molecules, and cell signals, one of the most important is the microvasculature [3], [4], [11], [16]–[20].

Several lines of evidence suggest that the microvasculature plays an important role in regulating the balance between proliferation and quiescence in NSCs. A number of *in vitro* studies have shown that endothelial cells (ECs) secrete soluble factors that stimulate NSC self renewal and promote neurogenesis [11], [16], [21]. These data are bolstered by observations of the intact microenvironment, which is highly vascularized by a network of capillaries. Using immunohistochemistry in conjunction with a panel of neural markers, several groups have shown that neural stem and progenitor cells in the murine adult brain are closely apposed to vessels [4], [13], [17], [22]. Closer examination reveals that the stem cells extend intricate processes that wrap around the vessels [4], [17], [22], and that migratory neuroblasts use the vessel network as a scaffold as they undergo chain migration [4], [7], [8], [23]–[25]. A variety of cell adhesion molecules and extracellular matrix components have now been implicated in maintaining this structural association [4], [13], [26], and disruption of this niche cytoarchitecture *in vivo* significantly increases neural stem and progenitor cell proliferation in the mouse [4], [26]. Other work has shown that changes in blood flow and hemodynamics in the SEZ can also differentially regulate NSC proliferation [27]–[30]. Together, these data suggest that blood vessels in the endogenous SEZ regulate adult neurogenesis by releasing factors that are carried in the circulating plasma or secreted directly by ECs. This is consistent with data in other stem cell niche microenvironments, where blood vessels and blood flow have also been shown to play an important role [3], [31]–[37].

However, our understanding of the microvasculature in the SEZ is incomplete. While a variety of studies have endeavored to map various aspects of the neural stem cell niche, including the association of NSCs with vessels [4], [13], [17], [22], a detailed anatomical map of the SEZ microvascular network itself has not yet been made. Initial observations have noted that vessel structure in the SEZ seems to be different from the cerebral cortex [17], so a more detailed analysis could lead to important insights about vascular specialization within the neural stem cell niche. Even more crucially, very little is known about blood flow in the SEZ and the effects it might have on the surrounding microenvironment. In this investigation, we therefore aimed to answer some of these questions using a variety of techniques to assess the anatomy and physiology of the SEZ in three dimensions (3D). Our results demonstrate the presence of a specialized microvascular domain in the SEZ defined by unique vessel structure and low rates of blood flow, and identify sites of hypoxia within the SEZ. These results have important implications for the role of the vasculature in the specialized SEZ microenvironment.

## Materials and Methods

### Mouse Strains and Ethics Statement

Experiments utilized female CD-1 outbred mice (Charles River) aged 2–4 months. All protocols were approved by the Institutional Animal Care and Use Committee (IACUC) at Baylor College of Medicine (assurance number A-3823-01). For surgical procedures, mice were anesthetized using 2,2,2-Tribromoethanol [500 mg/kg in PBS] (Sigma-Aldrich) delivered by IP injection, or isoflurane [3% in O_2_] (Baxter) delivered by tabletop vaporizer (VetEquip). All efforts were made to minimize suffering.

### Vessel Imaging and Morphometric Analysis

Anesthetized mice were injected intracardially with 200 µL PBS containing 70,000 MW lysine-fixable/tetramethylrhodamine-labeled dextran [0.5 mg/mL] (Life Technologies), EDTA [1.5 mg/mL] (IBI Scientific), and Heparin Sulfate [0.25 mg/mL] (Sigma-Aldrich). After 3 min, mice were sacrificed by decapitation, and heads were immersion fixed overnight at 4°C in paraformaldehyde [4% in PBS] (Sigma-Aldrich). SEZ preparations were then dissected whole as previously described [22], [38], and mounted flat on glass slides using silicone spacers (Research Products International Corp.) and Fluoromount-G (Southern Biotech). Finally, all SEZ flatmounts were imaged using a Zeiss LSM 510 META confocal microscope (Carl Zeiss).

For morphometric analysis, confocal images of flatmounts were processed with Imaris 7.4.2 (Bitplane), using the “Autodepth” filament function to trace vessel segments in the anterior SEZ. Coordinates of these vessel segments were then exported to MATLAB R2008a (MathWorks) and analyzed using custom-written software to measure vessel density, tortuosity, and angle (for source code, see Scripts S1, S2, S3, S4). Vessel density was defined as the percentage of volume occupied by vessels, measured as a function of the perpendicular distance from the ependymal wall. Adjusted tortuosity index of a given vessel segment was defined as T = (L−D)/D, where T = tortuosity, L = path length of the segment, and D = linear distance between beginning- and end-points of the segment. Vessel angle at a given point was defined as the angle between the ependymal wall and the tangent of the vessel at that point. We assumed the location of the ependymal wall based on our previously published observation that, on average, the vessel plexus in the anterior SEZ is located 10 µm below the ependymal layer [13].

Finally, measurements of vessel density, tortuosity, and angle were grouped based on distance from the ependymal wall, using 10 µm bins. Uniformity of each parameter across all depths was then tested using the non-parametric Kruskal-Wallis test. When a statistically significant difference (p<0.05) was found, measurements were pooled into two groups representing the SEZ (depth  = 0−20 µm) [13] and the striatum (depth >20 µm), and the groups were compared using the non-parametric Wilcoxon Rank Sum test. All statistical calculations were performed using R 2.10.1 (R Foundation for Statistical Computing).

### Microsphere Deposition

Microsphere deposition [39] was used to estimate regional cerebral blood flow rates. First, the femoral artery of an anesthetized mouse was surgically exposed. A segment of suture silk was then threaded beneath the artery and pulled tight to occlude the blood flow without causing damage. Next, the femoral artery was cannulated using stretched PE-10 tubing (Becton, Dickinson and Company). Periodically, Lidocaine HCl [5% in PBS] (Sigma-Aldrich) was washed over the vessel to prevent vasoconstriction. Following cannulation, a small volume of anticoagulant solution containing EDTA [1.5 mg/mL] and Heparin Sulfate [0.25 mg/mL] in PBS was injected into the artery to inhibit clotting. Finally, the heart was exposed, and a suspension of fluorescent 10 µm diameter FluoSpheres® (Life Technologies) in anticoagulant solution was injected into the left ventricle of the heart at a total volume of 150–200 µL. Since the majority of microspheres of this size are trapped in the capillaries on the first pass through the circulation, the resulting deposition rate should be insensitive to any differences in vessel structure between the SEZ and striatum. At the onset of microsphere injection, the suture was removed from the femoral artery, and the free-flowing blood from the cannula was collected into an eppendorf tube to serve as an arterial reference sample. The heart was allowed to recover and beat for ∼3 min after the injection before blood collection from the cannula was abruptly stopped and the mouse was sacrificed by decapitation.

Afterwards, the volume of the arterial reference sample was measured using a pipetman (VWR), and the total sphere count of the arterial reference sample was estimated using a hemocytometer (INCYTO). Next, SEZ flatmounts were prepared with the addition of a DAPI counterstain [10 µg/mL] (Life Technologies) before mounting. Tiled three-dimensional images of the left and right SEZ for each mouse were then collected using a Zeiss LSM 510 META confocal microscope and a motorized stage. Images were processed with Imaris 7.4.2, using the spot detection function to automatically locate each microsphere in three dimensions. The distance of each sphere from the ependymal wall could then be determined using the DAPI counterstain. Finally, the absolute rate of blood flow in each flatmount was determined by normalizing microsphere counts within the tissue to the microsphere count and flow rate of the corresponding arterial reference sample. The density of brain tissue was approximated to be 1.0 g/mL based on published measurements 40,41. In order to control for sufficient mixing of microspheres in the arterial circulation, we excluded any mouse in which the overall flow rate in either the left or right flatmount deviated from the overall bilateral flow rate by more than 15%.

Finally, blood flow measurements were grouped based on distance from the ependymal wall, using 10 µm bins. Uniformity of measured blood flow across all depths was then tested using the non-parametric Kruskal-Wallis test. When a statistically significant difference (p<0.05) was found, measurements were pooled into two groups representing the SEZ (depth  = 0−20 µm) and the striatum (depth >20 µm), and the groups were compared using the non-parametric Wilcoxon Rank Sum test. All statistical calculations were performed using R 2.10.1.

### Immunohistochemistry and Detection of Functional Hypoxia

For functionally measuring hypoxia, mice were administered an IP injection of Hypoxyprobe-1 [60 mg/kg in PBS] (HPI, Inc.), or PBS alone as a negative control [42]. After ∼30 min, mice were anesthetized and sacrificed by intracardial perfusion of paraformaldehyde [4% in PBS]. Final exposure time to Hypoxyprobe-1 was ∼35–40 min. Brains were either dissected for preparation of SEZ wholemounts, or sliced into 70 µm thick coronal sections using a vibratome (Leica), collecting all sections from 0 to 0.8 mm anterior to bregma in PBS.

For immunostaining, SEZ wholemounts were: postfixed with paraformaldehyde [4% in PBS] for 30 min; rinsed 3×10 min in PBS; permeabilized overnight in PBT [0.1% Triton X-100 in PBS] (Promega) at 4°C; blocked overnight in blocking solution [2% Donkey Serum in PBT] (Sigma-Aldrich) at 4°C; incubated overnight at 4°C with primary antibodies diluted in blocking solution; rinsed 3×10 min in blocking solution; incubated for 1.5 h at room temperature (RT) with secondary antibodies [1∶500] and DAPI [10 µg/mL] in blocking solution; rinsed 3×10 min in PBS; and then finally mounted as above.

Alternatively, coronal brain slices were: rinsed in PBS; permeabilized for 10 min at RT with 0.5% Triton X-100 in PBS; blocked 6–8 h at RT in Mouse Ig blocking reagent (Vector Laboratories); rinsed 2×2 min in PBS; incubated 5 min with MOM protein concentrate diluent [0.08% in PBS] (Vector Laboratories); incubated overnight at 4°C with primary antibodies diluted in MOM protein concentrate diluent [0.08% in PBS]; rinsed 3×10 min in PBS; incubated 1–2 h at RT with secondary antibodies [1∶500] and DAPI [10 µg/mL] in MOM protein concentrate diluent [0.08% in PBS]; rinsed 3×10 min in PBS; and then finally mounted on slides with Fluoromount-G. As necessary, NeuroTrace Fluorescent Nissl Stain [1∶50] (Life Technologies) was also added to the secondary antibody wash to identify neurons.

For list of primary antibodies, see Supporting Information (Table S1). Antibodies against Hypoxyprobe-1 were used to identify functional hypoxia. Secondary antibodies [1∶500] were all raised in donkey or goat and conjugated to Alexa Fluor® dyes (Life Technologies).

When necessary, linear unmixing was implemented using the Zeiss LSM 510 META microscope to eliminate spectral overlap between fluorochromes. Additional image analysis was performed using Imaris 7.4.2 for Gaussian filtering (when stated in figure legends) or for generating 3D reconstructions. Cell proximities to vessels were assessed by measuring distances between cell nuclei and the closest vessel surface, excluding cells that were closer to the boundaries of the z-stacks than to the nearest vessel. In any given treatment group, all adjustments to brightness and contrast were applied uniformly to every image in the group, and uniformly throughout each individual image.

## Results

### The SEZ Constitutes a Specialized Microvascular Domain

**Figure 1:**
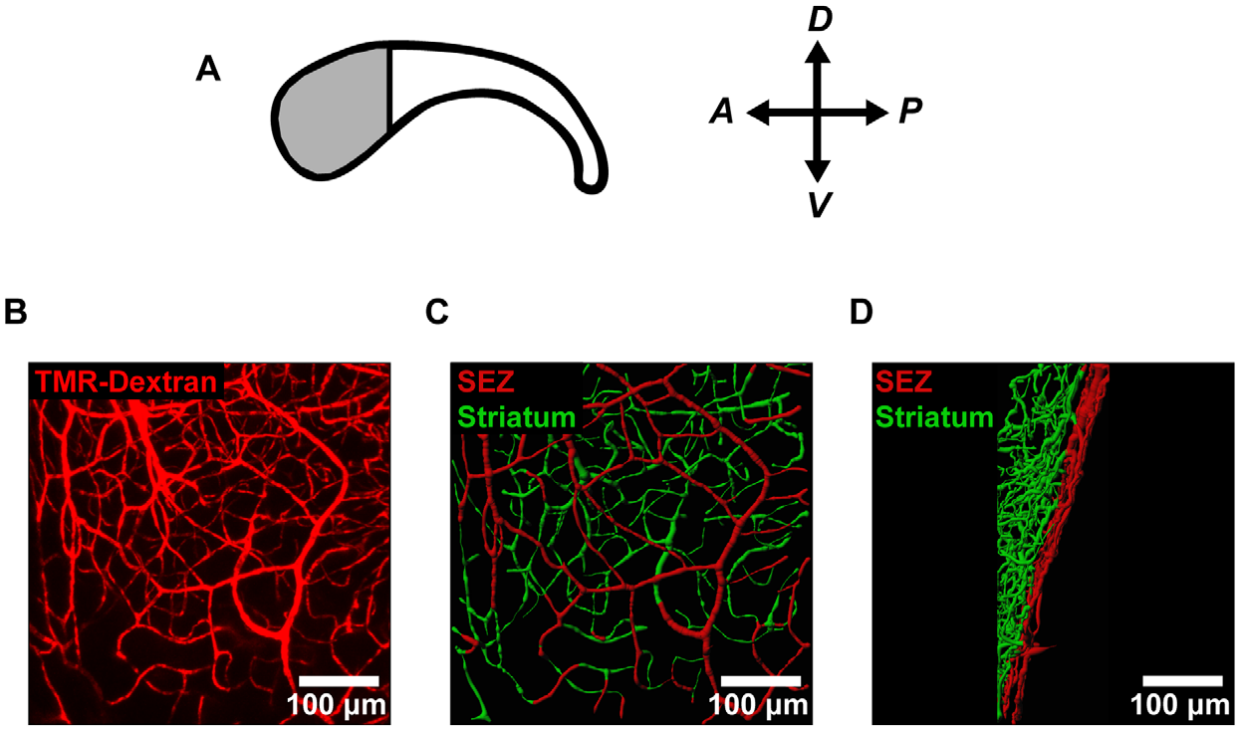
Confocal imaging reveals microvascular architecture in SEZ flatmounts. (**A**) For analysis of vessel structure in the neural stem cell niche, we focused on the anterior region of the SEZ (shaded region) where the density of neural stem cells is highest. (**B**) The microvessels in the anterior SEZ were labeled with an intracardial injection of fluorescent tetramethylrhodamine-labeled dextran (TMR-Dextran) and imaged *en-face*. (**C–D**) Vessel segments were traced using image processing software and color-coded according to distance from the ependymal wall (SEZ  = 0−20 µm; striatum >20 µm). Vessels are shown both *en-face* (C) and from the side (D).

To assess vascular structure in the mouse neural stem cell niche, we labeled the vessels by administering an intracardial injection of fluorescently labeled dextran. We focused our analysis on the anterior SEZ (Figure 1A), where the density of neural stem cells is highest [22]. This labeling technique enabled high-resolution imaging of the SEZ microvasculature (Figure 1B) and subsequent modeling of vessel structure in 3D (Figure 1C–D). We then used these 3D models to make morphometric measurements of vessels in the SEZ (depth  = 0−20 µm) and the adjacent striatum (depth >20 µm). This definition of the SEZ was used because SEZ neurogenesis is restricted to the first 20 µm beneath the ependymal wall [13]. Our images revealed striking differences between vessel architecture in these two regions, with the SEZ (shown in red) occupied by a planar plexus of relatively non-tortuous (straight) vessels, but the striatum (shown in green) occupied by a more disorganized network of tortuous vessels (Figure 1C–D). When we extended this analysis to more posterior portions of the SEZ, we observed similar trends in vessel structure (Figure S1).

**Figure 2:**
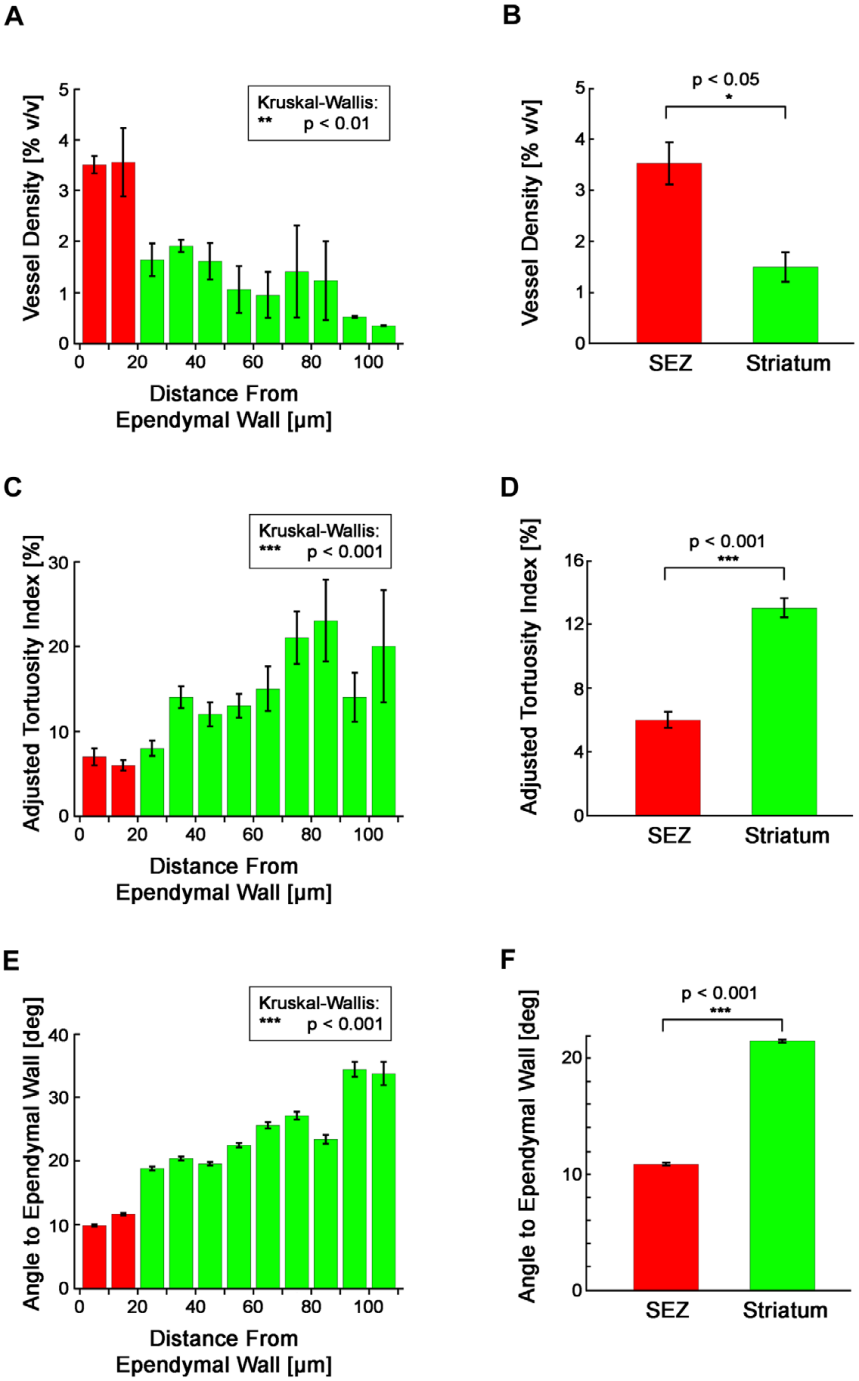
Vessels in the anterior SEZ and striatum are morphologically distinct. (**A**) Mean vessel density is not equal at all depths (p<0.01, Kruskal-Wallis test). (**B**) Mean vessel density in the SEZ is significantly different from the mean density in the striatum (p<0.05, Wilcoxon Rank Sum test; n_mice_ = 5, n_observations,SEZ_ = 5, n_observations,Str_ = 5). (**C**) Mean vessel tortuosity is not equal at all depths (p<0.001, Kruskal-Wallis test). (**D**) Mean vessel tortuosity in the SEZ is significantly different from the mean tortuosity in the striatum (p<0.001, Wilcoxon Rank Sum test; n_mice_ = 5, n_observations,SEZ_ = 395, n_observations,Str_ = 904). (**E**) The mean angle of the ependymal wall to vessels is not equal at all depths (p<0.001, Kruskal-Wallis test). (**F**) The mean angle of the ependymal wall to vessels in the SEZ is significantly different from the mean angle in the striatum (p<0.001, Wilcoxon Rank Sum test; n_mice_ = 5, n_observations,SEZ_ = 10,988, n_observations,Str_ = 20,159). (**A–F**) Plotted values represent mean ± standard error.

Morphometric analysis further elucidated the differences between the vessels in the anterior SEZ and striatum (Figure 2). Our measurements revealed an abrupt change in vessel architecture at a depth of 20 µm, a depth which coincides with the edge of the SEZ region (Figure 2A, C, E). This clear boundary demonstrated a natural separation of the microvascular network into the two layered domains of the SEZ and striatum. When we compared these two domains, we found that the SEZ exhibits higher vessel density (Figure 2B; p<0.05), lower vessel tortuosity (Figure 2D; p<0.001), and greater alignment between vessels and the ependymal wall (Figures 2F, S2; p<0.001). Together, these results demonstrate that the SEZ constitutes a specialized microvascular domain defined by unique vessel structure.

### The SEZ Exhibits Low Rates of Regional Blood Flow

**Figure 3:**
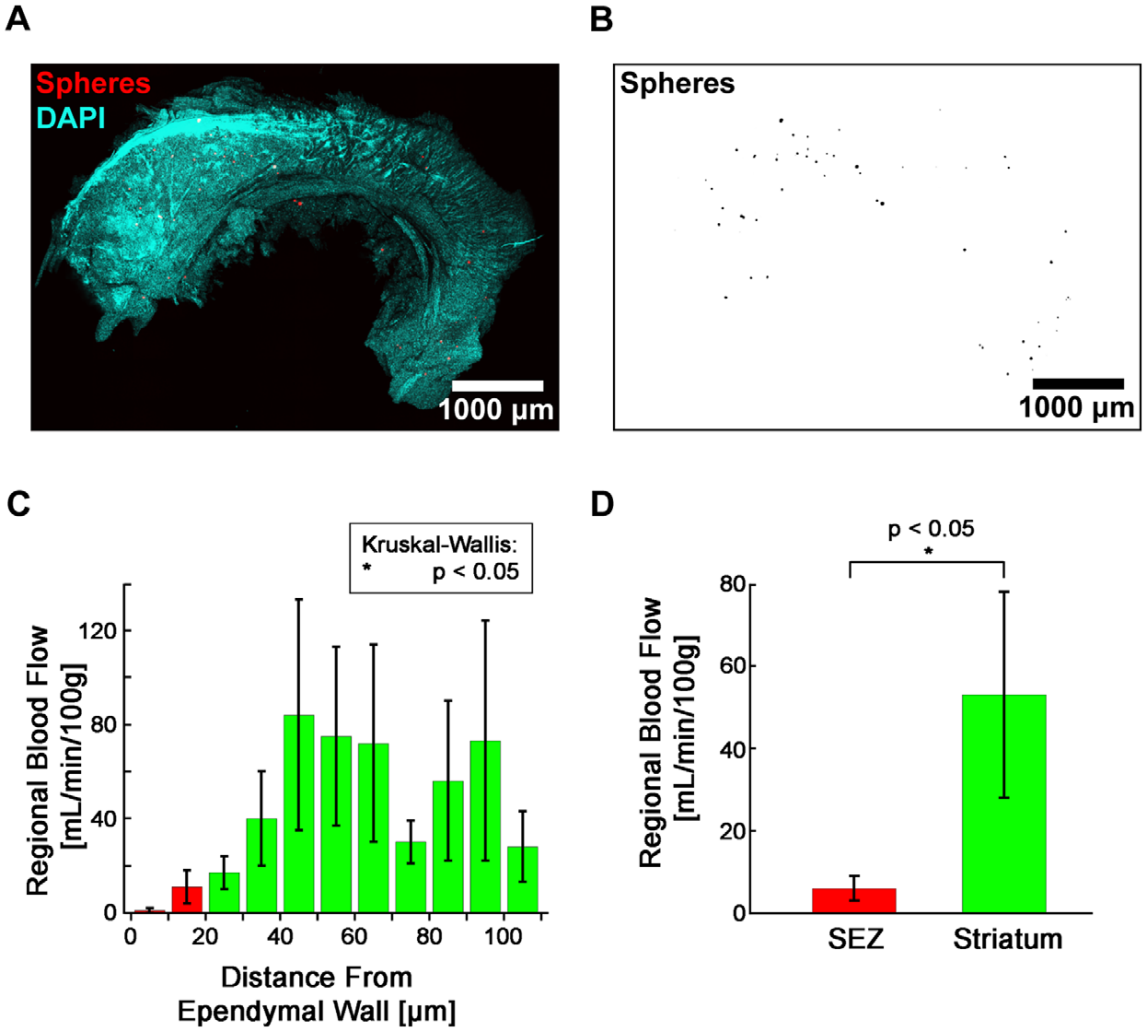
Regional blood flow in the SEZ is significantly less than in the striatum. (A–B ) Deposited microspheres were imaged in SEZ flatmounts for measuring regional blood flow. DAPI (A) was used to locate the surface of the ependymal wall for determining microsphere depths. (**C**) Mean blood flow is not equal at all depths (p<0.05, Kruskal-Wallis test). (**D**) Mean blood flow in the SEZ is significantly different from the mean blood flow in the striatum (p<0.05, Wilcoxon Rank Sum test; n_mice_ = 4, n_observations,SEZ_ = 8, n_observations,Str_ = 8). (**C–D**) Plotted values represent mean ± standard error.

Next, we went on to test if there were differences in blood flow to these two microvascular domains. Although blood flow in the SEZ is difficult to observe directly or in real time due to the region’s location deep within the brain and the small size of the capillaries, we were able to quantify blood flow in the SEZ using the well-validated method of microsphere deposition [39], [43]–[47]. By mixing 10 µm fluorescent microspheres with the arterial circulation, blood flow to a region of tissue can be measured by counting the number of microspheres that become lodged in its capillary bed (Figure 3A–B). With this method, we detected a non-uniform distribution of blood flow with respect to distance from the ependymal wall that was similar to what we detected with our morphometric measurements (Figure 3C, p<0.05). Overall, we measured a significantly lower mean blood flow rate in the SEZ (6 mL/min/100 g) as compared to the striatum (53 mL/min/100 g) (Figure 3D, p<0.05). These data demonstrated that the SEZ microvascular domain was distinct not only anatomically, but physiologically as well.

### Hypoxia is Detectable in Ependymal Cells and a Subpopulation of Neurons in the SEZ and Striatum

**Figure 4:**
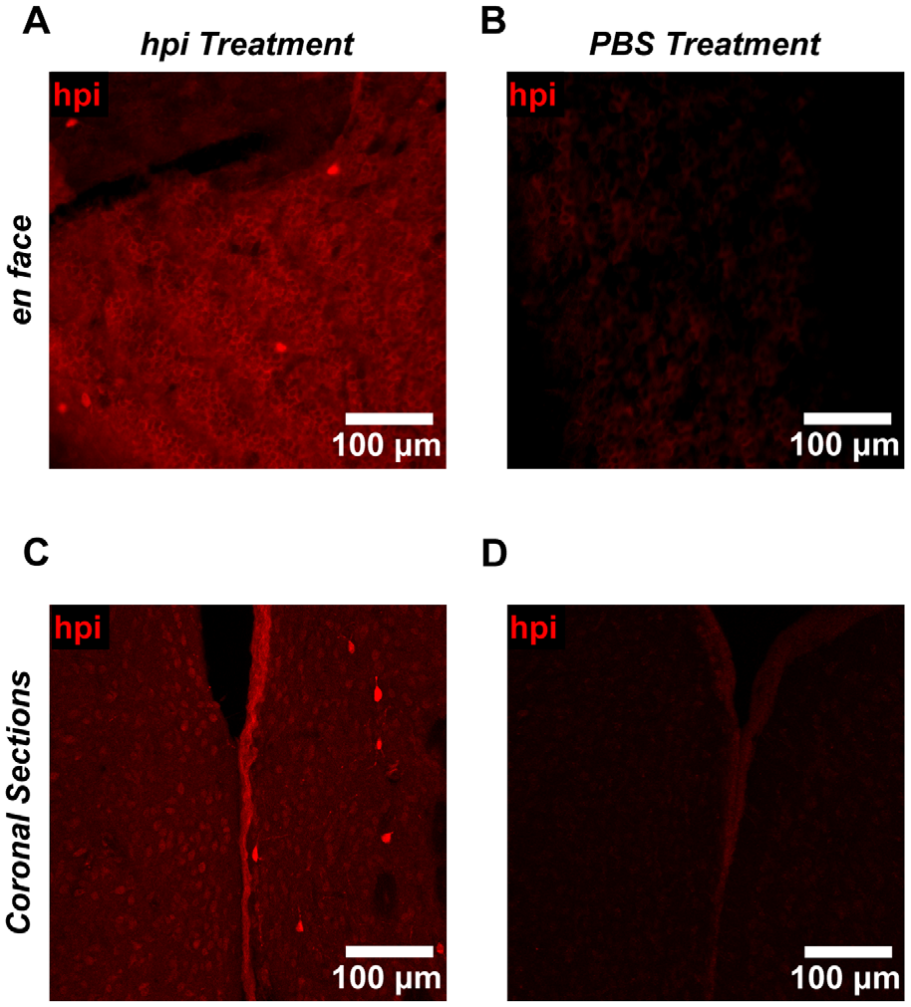
The neural stem cell niche exhibits functional hypoxia. (**A–B**) In mice treated with Hypoxyprobe-1 (hpi), hypoxia was detected in SEZ flatmounts throughout the ependymal cell layer (A). Bright Hypoxyprobe-1 staining was also detected in a subpopulation of cells immediately beneath the ependymal wall. Tissue treated with vehicle only (B) revealed minimal background staining, indicating that the staining seen in (A) is antigen specific. Immunostaining protocols and imaging settings were kept constant for (A) and (B). Representative images are shown. (**C–D**) In mice treated with Hypoxyprobe-1, hypoxia was also detected in the ependymal cell layer in coronal brain sections (C). Bright Hypoxyprobe-1 staining was detected in a distinct subpopulation of cells that was visible in the SEZ and striatum as well. Tissue treated with vehicle only (D) revealed minimal background staining, indicating that the staining seen in (C) is antigen specific. Immunostaining protocols and imaging settings were kept constant for (C) and (D). Representative images are shown.

**Figure 5:**
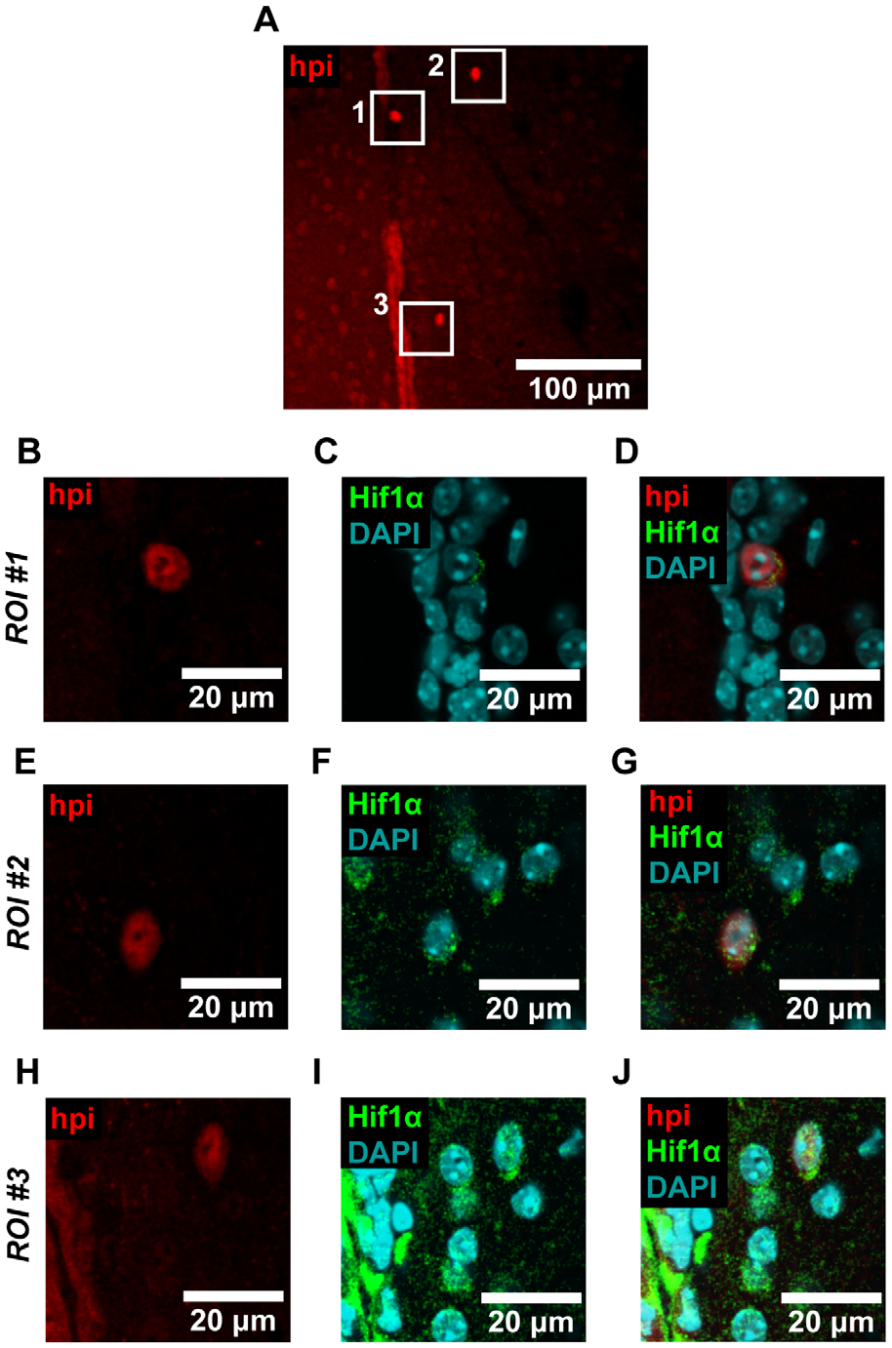
Hypoxyprobe-1 staining colocalizes with Hif1α. (**A**) Coronal brain sections were imaged, and regions of interest (ROIs) where Hypoxyprobe-1 (hpi) staining was observed were selected for closer inspection. (**B–J**) We observed colocalization between Hypoxyprobe-1 and Hif1α in each of the three ROIs shown, and in cells located in the SEZ, striatum, and ependymal layer. Overall, we observed colocalization with Hif1α in 83% of the cells that exhibited bright Hypoxyprobe-1 staining (n_cells_  = 18). Image data were processed with a Gaussian filter. Representative images are shown.

As an additional means of assessing vessel physiology in the SEZ, we assayed for hypoxia. To do this, we injected Hypoxyprobe-1 (HPI, inc.), a chemical tracer that identifies cells with low oxygen concentrations (pO_2_<10 mm Hg) [42]. We then detected this tracer with standard immunohistochemistry. Using this technique, we detected hypoxia in the ependymal layer that lines the wall of the lateral ventricle, as well as in a subpopulation of cells beneath this layer (Figure 4). This was evident both in SEZ flatmounts (Figure 4A–B) and in coronal brain sections (Figure 4C–D) by comparing Hypoxyprobe-1 treated tissue to vehicle-only treated controls. Immunostaining further revealed that Hypoxyprobe-1 staining colocalized with Hif1α in the SEZ, striatum, and ependymal layer (Figure 5). Overall, we observed colocalization with Hif1α in 83% of cells that showed bright Hypoxyprobe-1 staining (n_cells_ = 18). These results confirmed the utility of Hypoxyprobe-1 as a marker of hypoxia in the brain.

**Figure 6:**
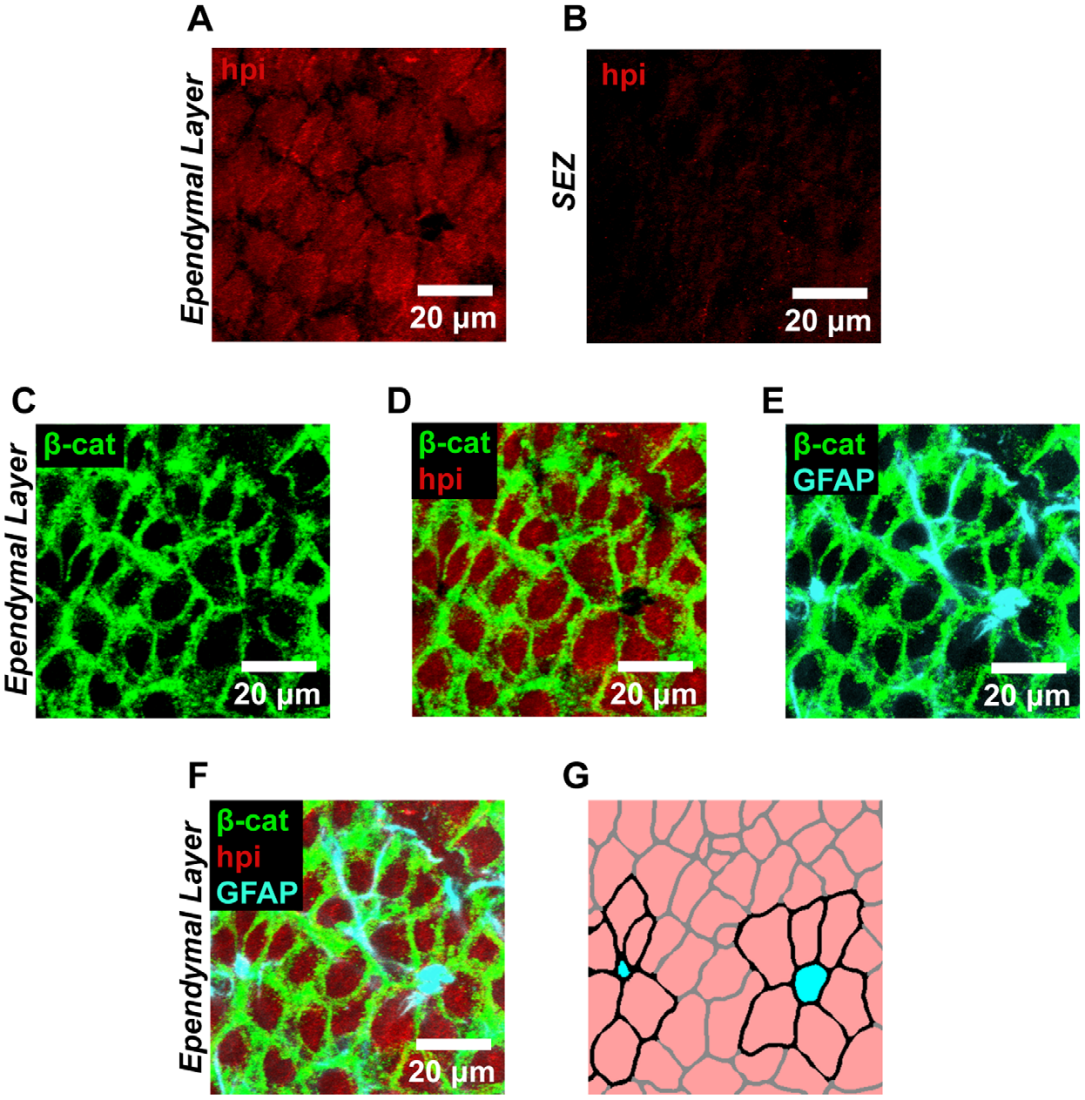
The ependymal cell layer in the SEZ is functionally hypoxic. (**A–B**) High magnification views of the ependymal cell layer (A) and underlying SEZ (B) confirmed that Hypoxyprobe-1 (hpi) staining was specific to the ependymal layer and not to the underlying tissue. (**C–D**) This observation was further verified by staining for β-catenin (C), which labels the cell boundaries of ependymal cells. Dual labeling (D) revealed that the ependymal cells did indeed exhibit Hypoxyprobe-1 staining. (**E–G**) Staining for GFAP (E) further confirmed this region as the neural stem cell niche by identifying neural stem cells within the ependymal wall that were arranged in a previously described pinwheel formation. Triple labeling revealed the presence of functional hypoxia throughout the neighboring ependymal cells (F). This is diagrammed in (G) where neural stem cells are shown in cyan, Hypoxyprobe-1 positive ependymal cells are shown in pink, and pinwheel structures are highlighted with cell borders drawn in black. (**A–F**) Linear unmixing was implemented to eliminate spectral overlap between fluorochromes. Representative images are shown.

We went on to examine hypoxia in SEZ flatmounts at high magnification (Figure 6). Comparison between cells at the surface of the ependymal wall (Figure 6A) and cells in the underlying tissue (Figure 6B) verified that hypoxia was specific to the most superficial layer of cells. Co-staining for β-catenin to label the cell membranes further identified these hypoxic cells as ependymal cells by revealing that they were arranged in an epithelial sheet at the boundary of the ventricle (Figure 6C–D). We also stained for GFAP to identify the apical surfaces of Type B1 neural stem cells and their surrounding pinwheel formations of cells (Figure 6E). Together with the other markers, this revealed that the apical surfaces of neural stem cells in the anterior SEZ are surrounded by hypoxic ependymal cells (Figure 6F–G).

**Figure 7:**
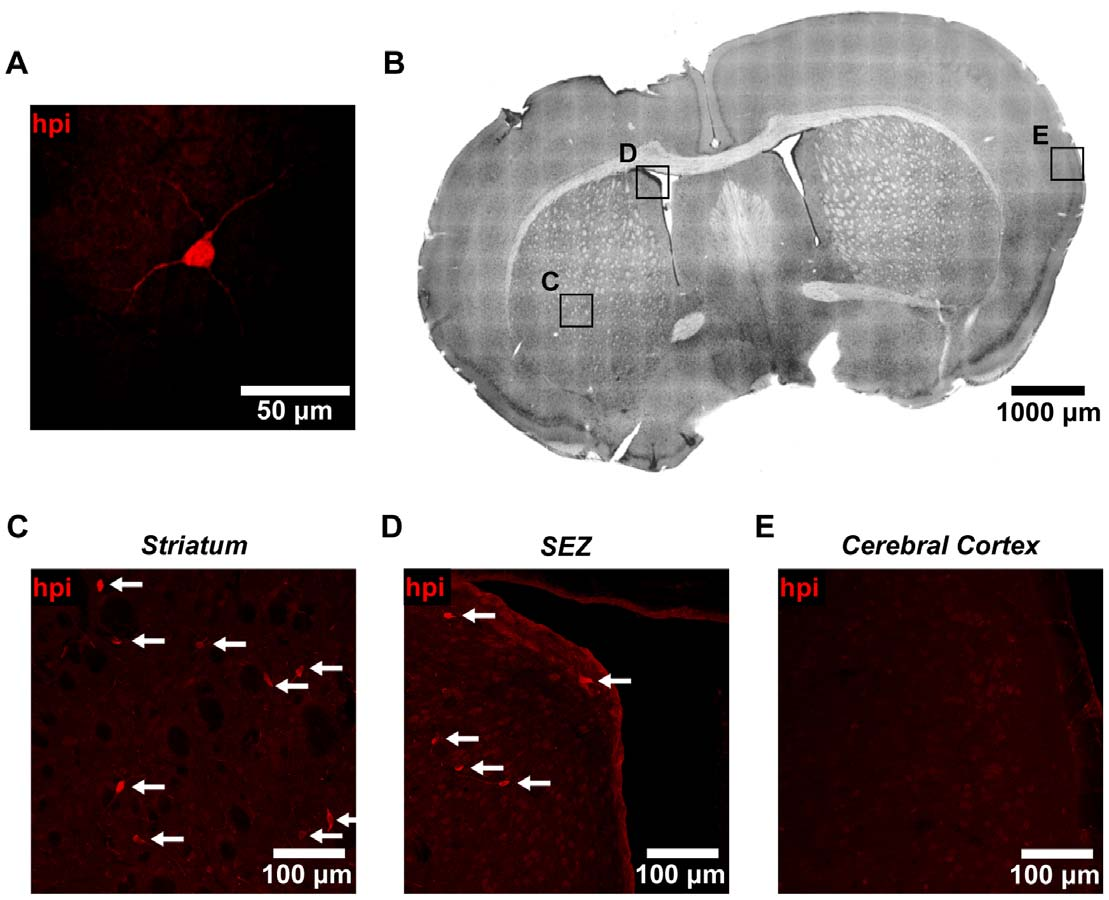
A subpopulation of non-ependymal cells in the SEZ and striatum exhibits functional hypoxia. (**A**) Non-ependymal cells staining brightly for Hypoxyprobe-1 (hpi) were characterized by large cell bodies and long dendrite-like processes. (**B**) Coronal brain sections were imaged to search for these hypoxic cells in various brain regions. Insets, shown at higher magnification in (C–E), are representative of the striatum (C), SEZ (D), and cerebral cortex (E). All insets were imaged using the same settings. (**C**) Cells staining brightly for Hypoxyprobe-1 (arrows) were observed in coronal sections of the striatum. Cells were always found in isolation, and never in clusters. (**D**) Non-ependymal cells staining brightly for Hypoxyprobe-1 (arrows) were also observed in coronal sections of the SEZ. Cells were always found in isolation, and never in clusters. (**E**) In comparison, cells staining brightly for Hypoxyprobe-1 were observed in the cerebral cortex only rarely.

**Figure 8:**
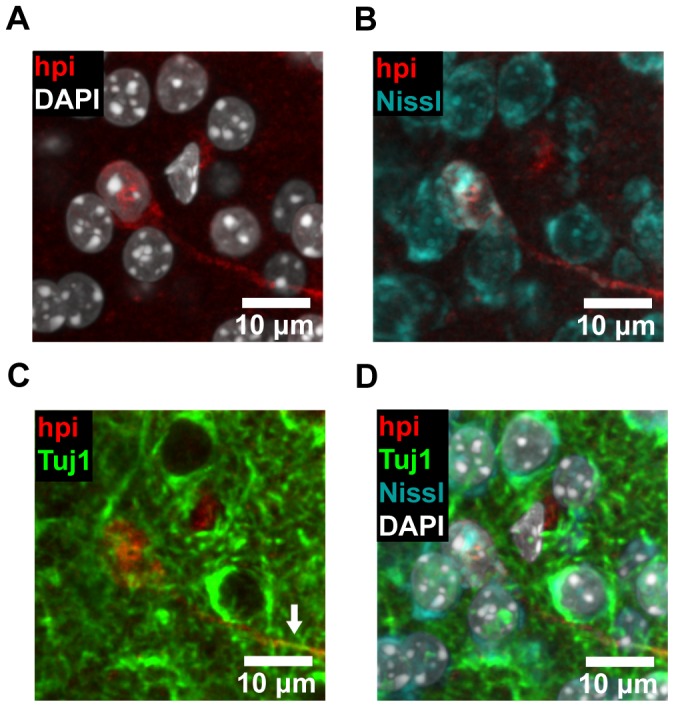
Hypoxic cells in the SEZ and striatum express neuronal markers. (**A–D**) Coronal brain slices were stained with Hypoxyprobe-1 (hpi) and neuronal markers. Hypoxyprobe-1 staining (A) was observed to colocalize with Nissl staining (B), a classic neuronal marker. The dendritic processes of cells positive for Hypoxyprobe-1 were also observed to express Tuj1 (β-tubulin-III), another neuronal marker (C, arrow). The merged image reveals the colocalization of all these markers (D). Image data were processed with a Gaussian filter. Representative images are shown.

Finally, we went on to further investigate the identity of the subpopulation of cells beneath the ependymal layer that stained brightly for Hypoxyprobe-1. These cells were characterized by large cell bodies and long dendrite-like processes (Figure 7A). Using coronal brain sections (Figure 7B), we found that these cells were located throughout the striatum and SEZ (Figure 7C–D, arrows), but that they were mostly absent from the cerebral cortex (Figure 7E). To define the molecular identity of these cells, we went on to use a wide panel of immunohistochemical markers. With this approach, we were able to identify these cells as differentiated neurons (Figure 8A–D), based on the colocalization of Hypoxyprobe-1 with Nissl staining (Figure 8B), and the expression of Tuj1 (β-tubulin-III) in dendrites (Figure 8C, arrow). Both of these markers are specific to neurons [11], [48], [49]. In contrast, we were unable to identify any hypoxic cells that expressed markers of glial, vascular, or peri-vascular cell types, nor were we able to identify Hypoxyprobe-1 positive neural stem or progenitor cells (Figure S3A–H) [4], [50]–[53]. These cells were also negative for markers of proliferation and apoptosis (Figure S3 I-J). Interestingly, these hypoxic neurons were found in isolation, rather than in clusters, and neighboring cells showed no evidence of hypoxia. Together, these data show that the SEZ is not in a state of global hypoxia. However, pronounced hypoxia is detectable in some of the cells that constitute the SEZ microenvironment, including the cells in the ependymal layer and a subset of neurons.

## Discussion

In this study, we have used a combination of techniques to identify an anatomically and physiologically distinct microdomain of blood vessels located in the murine SEZ neural stem cell niche. Due to the roles of the microvasculature in regulating adult neurogenesis [11],[17],[18], these observations could have important implications for our understanding of the SEZ neurogenic environment.

Our analysis of vessel structure led us to identify a specialized microvascular domain in the SEZ. This domain was characterized by relatively high vessel density, low vessel tortuosity, and vessels aligned mostly parallel to the ependymal wall. Notably, this domain occupied only the layer of tissue where neurogenesis is known to occur. Interestingly, high vessel density and organized vascular orientation has also been previously observed in the rostral migratory stream [25]. Such site-specific specialization is significant because of the effects that vessel structure can have on blood flow and hemodynamics, both of which are major regulators of the EC transcriptome [52], [54], [55]. The unique structure we observed in the SEZ vessel bed is thus likely to have a major impact on the secretion of angiocrine signals by ECs, and thereby a major impact on the microenvironment.

We were also able to identify physiological differences between the distinct microvascular domains in the SEZ and striatum. To do this, we used the established technique of microsphere deposition [39], [43], [47] to estimate rates of blood flow in each region. These measurements demonstrated that blood flow depended on distance from the ependymal wall in a way that mirrored what we observed for vessel morphology. Specifically, while our measurements of blood flow in the striatum were similar to published estimates of blood flow in the striatum and cerebral cortex [56], [57], our measurements in the SEZ were significantly lower, with a mean of 6 mL/min/100 g. This low measurement of perfusion in a region of relatively high vessel density suggests that blood flow in the SEZ is limited somewhere further upstream in the vascular tree. These results support the idea that the different microvascular domains in the SEZ and striatum differ not only in structure but also in function. Furthermore, this reveals low-flow conditions within the SEZ that likely have important implications for the niche environment. There is evidence that other adult stem cell niche environments also receive low levels of blood flow [36], [58], [59], and it has been hypothesized that this may be an important means of promoting stem cell quiescence in the adult. Consistent with this, others have shown that global changes in cerebral blood flow can perturb SEZ neurogenesis, even in the absence of ischemia [27]–[30]. Disruption of the low flow conditions in the SEZ by distal ischemic infarcts could therefore be one mechanism by which stroke is able to non-locally induce compensatory neural stem cell proliferation in the SEZ [60].

Finally, we concluded our study of microvascular physiology in the SEZ by testing for hypoxia. Hypoxia and redox status have been implicated as regulators of stem cell function in a variety of different stem cell types, including neural stem cells [26], [36],[58],[59],[61]–[65]. Accordingly, we wanted to assess if functional hypoxia was detectable in the SEZ. We did this using Hypoxyprobe-1, a molecular tracer of pronounced hypoxia (pO_2_<10 mm Hg) [42], which we validated using Hif1α as a second marker for hypoxia. With these techniques, we were unable to detect hypoxia either in NSCs or globally throughout the SEZ, consistent with a previous study [63]. However, we did observe hypoxia in the ependymal layer that lines the ventricles. This is potentially due both to the region’s low blood perfusion, and its proximity to the avascular ventricle. The ependymal cells that make up this layer help maintain the structural integrity of the niche, and serve as an important source of paracrine signals that regulate neurogenesis in the SEZ microenvironment [22], [26], [66]. Furthermore, although ependymal cells are not thought to be *bona fide* stem cells, there is some controversial evidence that they are capable of reentering the cell cycle to produce neurons [2], [67]–[70]. Interestingly, recent work has also shown that in the spinal cord, the ependymal cells actually are stem cells [71], [72], and that proliferation and differentiation in these cells is regulated by hypoxia [73], [74]. Future work will be needed to determine whether the hypoxia we observed in SEZ ependymal cells might play an analagous role in regulating paracrine signaling or cell cycle reentry.

We also identified a population of non-ependymal cells in the SEZ that exhibited high levels of hypoxia. These cells were consistently seen in the SEZ, as well as in the striatum, but only rarely in the cerebral cortex. Additionally, this cell population appeared to be exclusively composed of differentiated neurons, based on their expression of neuronal markers and consistent morphology. Since the neurons identified by Hypoxyprobe-1 were in well-vascularized regions and always found in isolation, it is possible that the cause of hypoxia in these cells is cell-intrinsic. Elevated cell metabolism is capable of depleting intracellular oxygen levels [75], [76], and so one potential explanation is that these cells are unusually metabolically active as compared to their neighbors. Another possibility is that these hypoxic cells are oxygen-sensing neurons that help regulate the response of the central nervous system to global changes in oxygen levels [77]. However, future studies will be necessary to distinguish between these possibilities and define the function of these cells.

In summary, this work helps elucidate the contributions made by blood vessels, blood flow, and hypoxia to the specialized microenvironment of the murine neural stem cell niche. We have identified a specialized microvascular domain specific to the SEZ defined by unique vessel architecture and low rates of blood flow. Furthermore, we have shown that the SEZ exhibits hypoxic conditions within the ependymal layer and in a subset of resident neurons. These findings further implicate the vasculature as an important determinant of the specialized environment of the SEZ, and highlight important questions about the role of vessel physiology in the adult neural stem cell niche. To answer these questions, learning more about the close relationship between vessel structure and function, and how this relationship is regulated *in vivo*, will be essential.

## Supporting Information

Figure S1 **Vessel structure along the anterior-posterior axis of the SEZ. (A)** Microvessels were labeled with an intracardial injection of fluorescent tetramethylrhodamine-labeled dextran (TMR-Dextran) and imaged *en-face*. Vessel structure in the SEZ and striatum was assessed along the anterior-posterior axis. Representative images from each shaded region are shown. **(B–G)** Differences between vessels in the SEZ (B–D) and striatum (E–G) were similar throughout the anterior-posterior axis of the flatmounted tissue.
(TIF)

Figure S2 **Vessel orientations in the SEZ and striatum are distinct.** The distribution of vessel angles to the ependymal wall is significantly different for the SEZ than for the striatum (p<0.001, Kolmogorov-Smirnov test; n_mice_ = 5, n_observations,SEZ_ = 10,988, n_observations,Str_ = 20,159).
(TIF)

Figure S3 **Cell-type markers not expressed by Hypoxyprobe-1 positive cells. (A–J)** Cells that stained brightly for Hypoxyprobe-1 (hpi) were screened for the expression of a variety of markers. Shown are representative images of immunostained coronal sections (A, C-J) and SEZ wholemounts imaged *en face* (B). Cells were tested for markers of endothelial cells [Pecam1] (A–B), pericytes [NG2] (C), microglia [F4/80] (D), oligodendrocytes [NG2 and Olig2] (C, E), astroglia [GFAP] (F), and neural stem and progenitor cells [GFAP, Mash1, and Dcx] (F–H). Cells were also tested for markers of proliferation [Ki67] (I) and apoptosis [Cleaved Caspase-3] (J). Hypoxyprobe-1 positive cells did not express any of these markers. Image data in (B) were processed with a Gaussian filter.
(TIF)

Table S1 **List of primary antibodies.** These primary antibodies were used for all immunohistochemistry experiments.
(DOC)

Script S1 **MATLAB script for splitting vessel networks.** What follows is a MATLAB script that uses a reference plane to divide a network of vessels into two separate networks. Vessel networks are defined as filament coordinates, using the format implemented by Imaris 7.4.2 (Bitplane). Filament coordinates can be exported using ImarisXT. This script was used to divide vessels in the SEZ from vessels in the striatum.
(PDF)

Script S2 **MATLAB script for calculating vessel tracks.** What follows is a MATLAB script that takes filament coordinates exported by Imaris 7.4.2 (Bitplane), and defines vessel tracks that connect branch points to branch points, branch points to end points, or end points to end points. Filament coordinates can be exported using ImarisXT. Each of the calculated vessel tracks defines a vessel unit that can be used for calculating vessel tortuosity, vessel angle (with respect to a reference plane), or vessel density (with respect to a reference plane).
(PDF)

Script S3 **MATLAB script for calculating vessel tortuosity.** What follows is a MATLAB script that takes filament coordinates exported by Imaris 7.4.2 (Bitplane) and the tracks calculated by “calctracks.m”, and calculates vessel tortuosity for each track. Filament coordinates can be exported using ImarisXT.
(PDF)

Script S4 **MATLAB script for plotting vessel networks and calculating vessel density or angle with respect to a reference plane.** What follows is a MATLAB script that plots a vessel network using exported filament coordinates from Imaris 7.4.2 (Bitplane) and the tracks calculated by “calctracks.m”. Filament coordinates can be exported using ImarisXT. The script can then use the plotted vessel network to calculate vessel density or angle with respect to a reference plane.
(PDF)
