# Supplementary figures and images for: A Specialized Microvascular Domain in the Mouse Neural Stem Cell Niche
Source: PLoS One. 2013 Jan 7;8(1):e53546. doi: 10.1371/journal.pone.0053546 (PMC3538546; doi:10.1371/journal.pone.0053546)

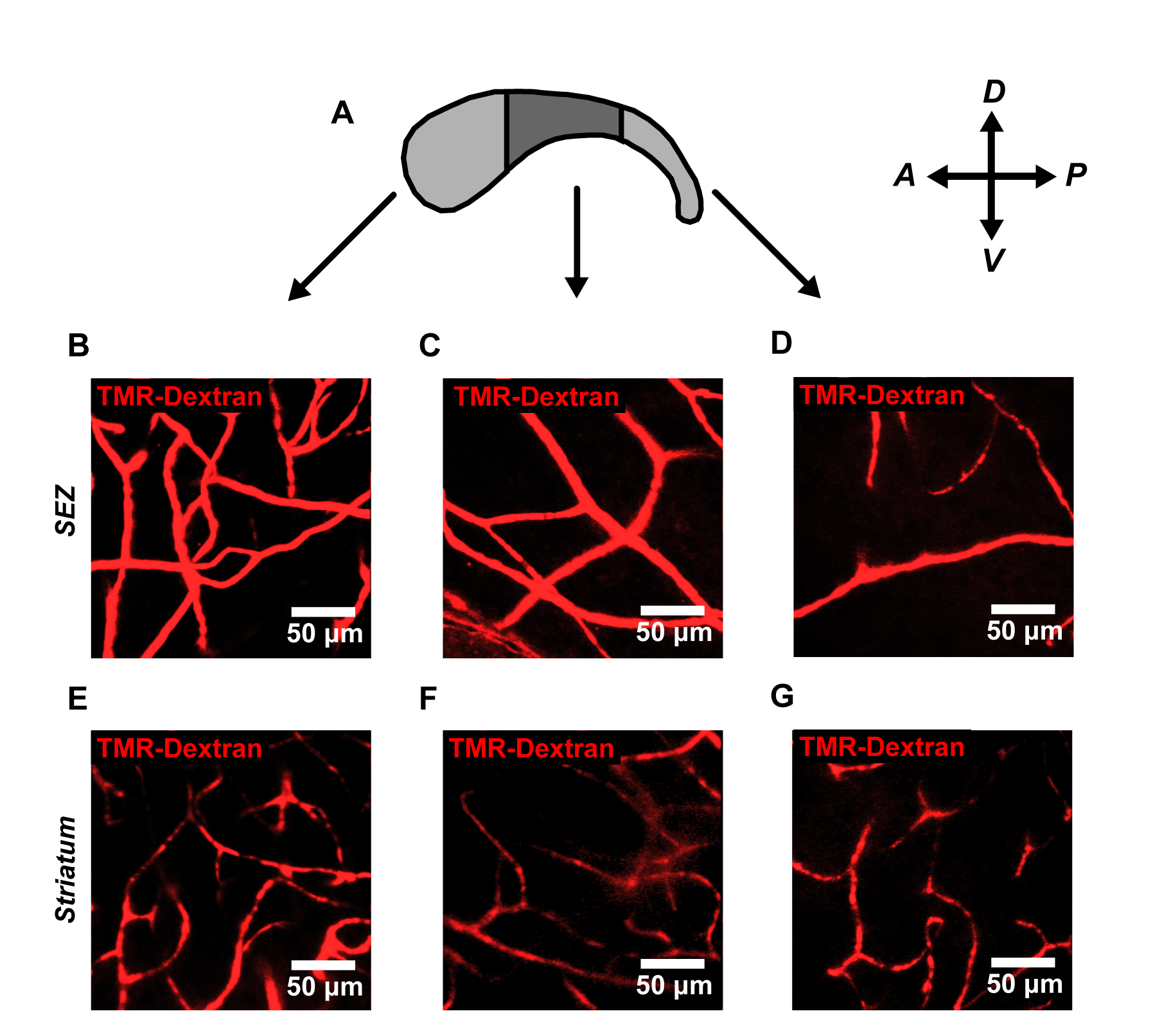

Supplement: Figure S1 — Vessel structure along the anterior-posterior axis of the SEZ. (A) Microvessels were labeled with an intracardial injection of fluorescent tetramethylrhodamine-labeled dextran (TMR-Dextran) and imaged en-face. Vessel structure in the SEZ and striatum was assessed along the anterior-posterior axis. Representative images from each shaded region are shown. (B–G) Differences between vessels in the SEZ (B–D) and striatum (E–G) were similar throughout the anterior-posterior axis of the flatmounted tissue. (TIF) [file pone.0053546.s001.tif]

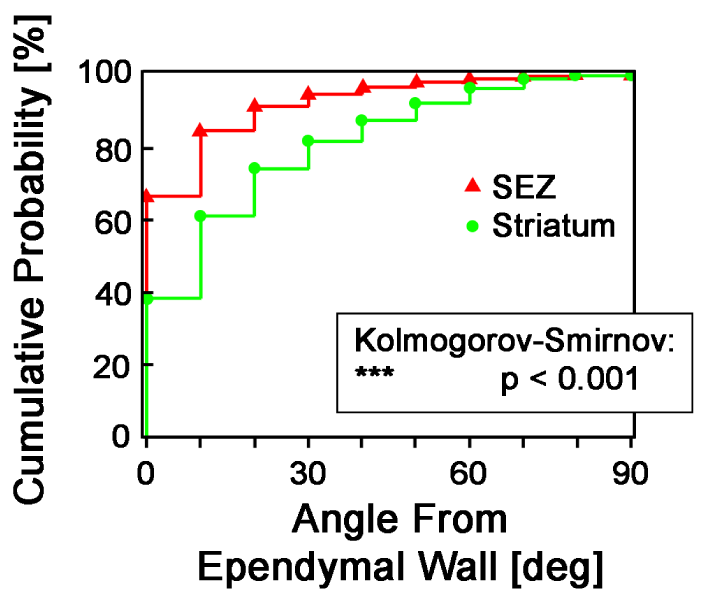

Supplement: Figure S2 — Vessel orientations in the SEZ and striatum are distinct. The distribution of vessel angles to the ependymal wall is significantly different for the SEZ than for the striatum (p<0.001, Kolmogorov-Smirnov test; nmice = 5, nobservations,SEZ = 10,988, nobservations,Str = 20,159). (TIF) [file pone.0053546.s002.tif]

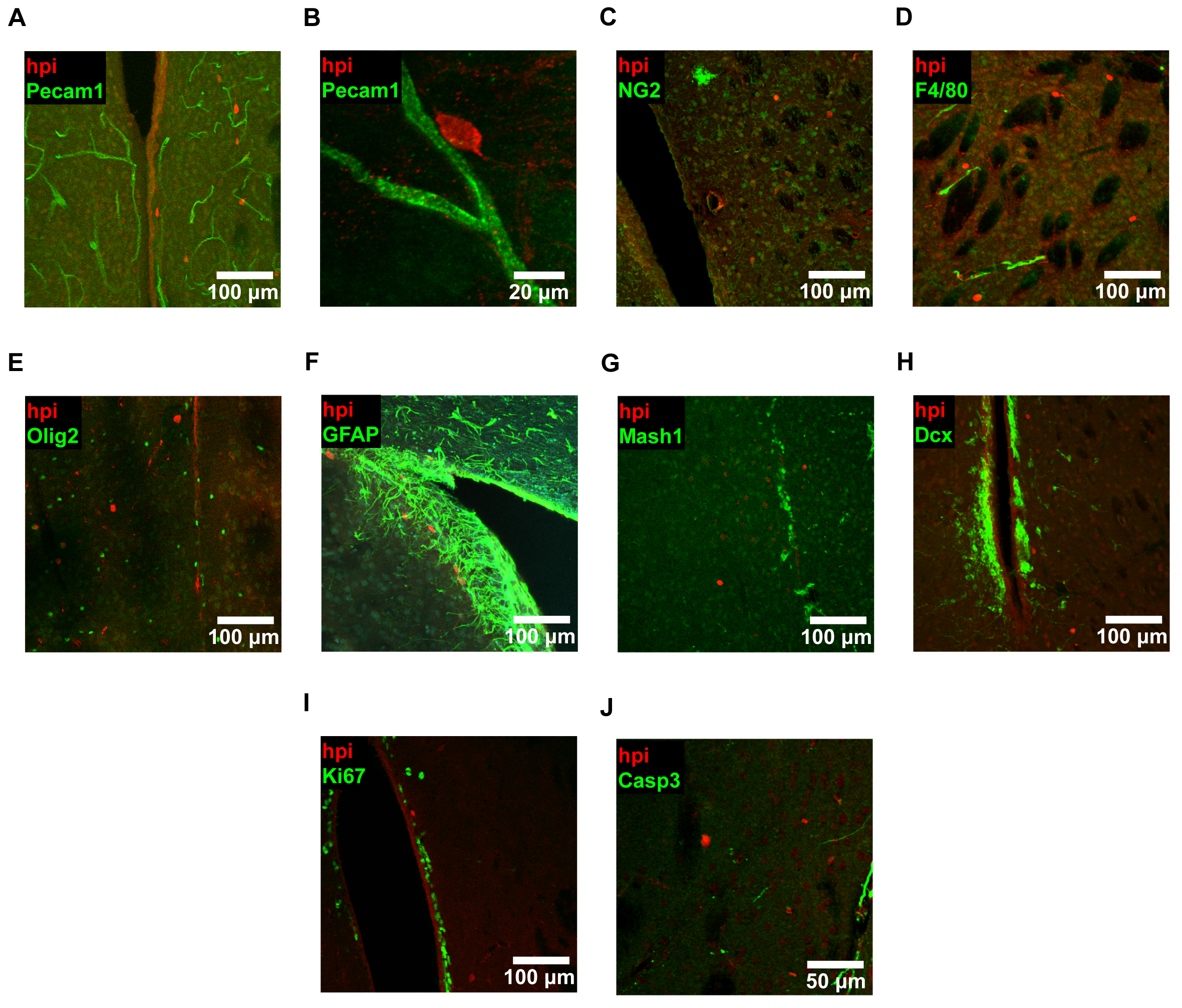

Supplement: Figure S3 — Cell-type markers not expressed by Hypoxyprobe-1 positive cells. (A–J) Cells that stained brightly for Hypoxyprobe-1 (hpi) were screened for the expression of a variety of markers. Shown are representative images of immunostained coronal sections (A, C-J) and SEZ wholemounts imaged en face (B). Cells were tested for markers of endothelial cells [Pecam1] (A–B), pericytes [NG2] (C), microglia [F4/80] (D), oligodendrocytes [NG2 and Olig2] (C, E), astroglia [GFAP] (F), and neural stem and progenitor cells [GFAP, Mash1, and Dcx] (F–H). Cells were also tested for markers of proliferation [Ki67] (I) and apoptosis [Cleaved Caspase-3] (J). Hypoxyprobe-1 positive cells did not express any of these markers. Image data in (B) were processed with a Gaussian filter. (TIF) [file pone.0053546.s003.tif]
