# Supplementary material for: A Specialized Microvascular Domain in the Mouse Neural Stem Cell Niche
Source: PLoS One. 2013 Jan 7;8(1):e53546. doi: 10.1371/journal.pone.0053546 (PMC3538546; doi:10.1371/journal.pone.0053546)
Supplement: Table S1 — List of primary antibodies. These primary antibodies were used for all immunohistochemistry experiments. (DOC) [file pone.0053546.s004.doc]

| **Antigen** | **Host** | **Dilution** | **Company** | **Catalog #** |
| --- | --- | --- | --- | --- |
| Beta-catenin | Rabbit | 1:100 | Cell Signaling | 9587 |
| Beta-tubulin-III | Rabbit | 1:250 | Covance | MRB-435P |
| Cleaved Caspase-3 | Rabbit | 1:200 | Cell Signaling | 9661S |
| Dcx | Guinea Pig | 1:500 | Millipore | AB5910 |
| F4/80 | Rat | 1:100 | Caltag Laboratories | MF48000 |
| GFAP | Rabbit | 1:100 | DAKO | Z0334 |
| GFAP | Rat | 1:50 | Life Technologies | 13-0300 |
| Hif1-alpha | Mouse | 1:50 | AbCam | ab16066 |
| Hypoxyprobe-1 (hpi) | Mouse | 1:50 | HPI, Inc. | HP1-100Kit |
| Hypoxyprobe-1 (hpi) | Rabbit | 1:100 | HPI, Inc. | HP3-100Kit |
| Ki67 | Rabbit | 1:50 | AbCam | ab16667 |
| Mash1 | Mouse | 1:100 | BD Pharmingen | 556604 |
| NG2 | Rabbit | 1:50 | Millipore | AB5320 |
| Olig2 | Rabbit | 1:100 | Millipore | AB9610 |
| Pecam1 | Rat | 1:50 | BD Pharmingen | 550274 |
