# Supplementary material for: A Specialized Microvascular Domain in the Mouse Neural Stem Cell Niche
Source: PLoS One. 2013 Jan 7;8(1):e53546. doi: 10.1371/journal.pone.0053546 (PMC3538546; doi:10.1371/journal.pone.0053546)
Supplement: Script S1 — MATLAB script for splitting vessel networks. What follows is a MATLAB script that uses a reference plane to divide a network of vessels into two separate networks. Vessel networks are defined as filament coordinates, using the format implemented by Imaris 7.4.2 (Bitplane). Filament coordinates can be exported using ImarisXT. This script was used to divide vessels in the SEZ from vessels in the striatum. (PDF) [file pone.0053546.s005.pdf]

```
function output = splitfilament

% splitfilament.m
%
% Copyright 2010-2012,
% Baylor College of Medicine
% All Rights Reserved
%
% Last modified 7/8/2012
%
% Uses a reference plane to divide a network of vessels into two separate
% networks. Vessel networks are defined as filament coordinates, using the
% format implemented by Imaris 7.4.2 (Bitplane). Filament coordinates can
% be exported using ImarisXT.
%
% Designed to use the margin between the SEZ and the Striatum in order to
% separate the two vessel networks, allowing for separate morphometric
% analyses.
%
% Assumes the reference plane is located at a depth of 10um below the
% ependymal wall, and that the SEZ is 20um thick.
%
%    Inputs:
%    -Directory containing filament coordinates that define reference plane
%    -Directory containing vessel coordinates
%
%    Outputs (one each for the SEZ, the Striatum, and the combination):
%    -Filament coordinates for each subset of vessels (includes
%    vFilamentXYZ, vFilamentRadius, vFilamentEdges, and vFilamentTypes).
%    Each set is saved into its own subdirectory of the parent directory
%    containing the vessel filament coordinates.
%


% Define directory containing vFilamentXYZ for filaments defining reference plane
directory_name1 = uigetdir('','Select Directory Containing filaments that define the↙
reference plane (exported from Imaris)')
cd(directory_name1)
vFilamentXYZ=csvread('vFilamentXYZ.txt');
Xcolv = vFilamentXYZ(:,1);
Ycolv = vFilamentXYZ(:,2);
Zcolv = vFilamentXYZ(:,3);
Const = ones(size(Xcolv));
Coefficients = [Xcolv Ycolv Const]\Zcolv; % Find the coefficients
XCoeff = Coefficients(1); % X coefficient
YCoeff = Coefficients(2); % X coefficient
CCoeff = Coefficients(3); % constant term
clear Coefficients Const Xcolv Ycolv Zcolv

% Define directory containing vFilamentXYZ, vFilamentRadius,
% vFilamentEdges, and vFilamentTypes for filaments to be split
directory_name2 = uigetdir('','Select Directory Containing filaments to be split (exported↙
```

```
from Imaris)')
cd(directory_name2)
vFilamentXYZ=csvread('vFilamentXYZ.txt');
vFilamentRadius=csvread('vFilamentRadius.txt');
vFilamentEdges=csvread('vFilamentEdges.txt');
vFilamentTypes=csvread('vFilamentTypes.txt');

mkdir(directory_name2,'Combined')
mkdir(directory_name2,'SEZ')
mkdir(directory_name2,'Striatum')
csvwrite(strcat(directory_name2,'\Combined','\vFilamentEdges.txt'),vFilamentEdges);
csvwrite(strcat(directory_name2,'\Combined','\vFilamentTypes.txt'),vFilamentTypes);
csvwrite(strcat(directory_name2,'\Combined','\vFilamentRadius.txt'),vFilamentRadius);
csvwrite(strcat(directory_name2,'\Combined','\vFilamentXYZ.txt'),vFilamentXYZ);

% Calculate distance between each point and the reference plane. Formula
% for distance calculated by solving for the equation of a line that lies
% normal to the reference plane and intersects with the given point.
WallPtDist=((vFilamentXYZ(:,1).*XCoeff)+(vFilamentXYZ(:,2).*YCoeff)-(vFilamentXYZ(:,3))+↙
(CCoeff));
WallPtDist=(WallPtDist)./(sqrt((XCoeff^2)+(YCoeff^2)+1));
plot(WallPtDist)
mean(WallPtDist)

% Defines SEZ thickness as 20um, and determines which side of reference
% plane contains striatum

thickness=20;
halfthick=thickness/2;
if mean(WallPtDist)<0
    SEZPts=WallPtDist>(-1*halfthick);
else
    SEZPts=WallPtDist<(halfthick);
end

% Split filaments. If any points in a segment belong to the striatum, then
% the entire segment is classified as striatum.
edgetest=ismember(vFilamentEdges,find(~SEZPts));
edgetest2=edgetest(:,1)+edgetest(:,2);
StrEdges=vFilamentEdges((find(edgetest2)),:);
SEZEdges=vFilamentEdges((find(edgetest2==0)),:);
vFilamentIndex=cumsum((vFilamentXYZ(:,1).*0)+1);
SEZXYZ=vFilamentXYZ((find(ismember(vFilamentIndex,SEZEdges))),:);
SEZTypes=vFilamentTypes((find(ismember(vFilamentIndex,SEZEdges))),:);
SEZRadius=vFilamentRadius((find(ismember(vFilamentIndex,SEZEdges))),:);
StrXYZ=vFilamentXYZ((find(ismember(vFilamentIndex,StrEdges))),:);
StrTypes=vFilamentTypes((find(ismember(vFilamentIndex,StrEdges))),:);
StrRadius=vFilamentRadius((find(ismember(vFilamentIndex,StrEdges))),:);
SEZIndAdjust=vFilamentIndex-(cumsum(~ismember(vFilamentIndex,SEZEdges)));
StrIndAdjust=vFilamentIndex-(cumsum(~ismember(vFilamentIndex,StrEdges)));
testsize=size(vFilamentIndex);
indmax=testsize(1);
```

```
for i=1:indmax
    SEZEdges(find(SEZEdges==i))=SEZIndAdjust(i);
end
for i=1:indmax
    StrEdges(find(StrEdges==i))=StrIndAdjust(i);
end

% Save the SEZ only filaments

csvwrite(strcat(directory_name2,'\SEZ','\vFilamentEdges.txt'),SEZEdges);
csvwrite(strcat(directory_name2,'\SEZ','\vFilamentTypes.txt'),SEZTypes);
csvwrite(strcat(directory_name2,'\SEZ','\vFilamentRadius.txt'),SEZRadius);
csvwrite(strcat(directory_name2,'\SEZ','\vFilamentXYZ.txt'),SEZXYZ);

% Save the Striatum only filaments

csvwrite(strcat(directory_name2,'\Striatum','\vFilamentEdges.txt'),StrEdges);
csvwrite(strcat(directory_name2,'\Striatum','\vFilamentTypes.txt'),StrTypes);
csvwrite(strcat(directory_name2,'\Striatum','\vFilamentRadius.txt'),StrRadius);
csvwrite(strcat(directory_name2,'\Striatum','\vFilamentXYZ.txt'),StrXYZ);

output='Finished Splitting Filaments';
```
