# Supplementary material for: A Specialized Microvascular Domain in the Mouse Neural Stem Cell Niche
Source: PLoS One. 2013 Jan 7;8(1):e53546. doi: 10.1371/journal.pone.0053546 (PMC3538546; doi:10.1371/journal.pone.0053546)
Supplement: Script S2 — MATLAB script for calculating vessel tracks. What follows is a MATLAB script that takes filament coordinates exported by Imaris 7.4.2 (Bitplane), and defines vessel tracks that connect branch points to branch points, branch points to end points, or end points to end points. Filament coordinates can be exported using ImarisXT. Each of the calculated vessel tracks defines a vessel unit that can be used for calculating vessel tortuosity, vessel angle (with respect to a reference plane), or vessel density (with respect to a reference plane). (PDF) [file pone.0053546.s006.pdf]

```
function calctracks

% calctracks.m
%
% Copyright 2010-2012,
% Baylor College of Medicine
% All Rights Reserved
%
% Last modified 7/8/2012
%
% Takes filament coordinates exported by Imaris 7.4.2 (Bitplane), and
% defines vessel tracks that connect branch points to branch points,
% branch points to end points, or end points to end points. Filament
% coordinates can be exported using ImarisXT.
%
% Each of the exported vessel tracks defines a vessel unit that is needed
% later by "calctortuosity.m" for calculating tortuosity, or by
% "vesselrender.m" for plotting vessels, calculating vessel angles w.r.t.
% a reference plane, or calculating density w.r.t. a reference plane.
%
%    Inputs:
%    -Directory containing vessel coordinates
%
%    Output:
%    "tracks.txt" - csv formatted file containing a matrix that lists each
%    track by column. For track k, the first point of the track is listed
%    at Row 1, Col k, the second point is listed at Row 2, Col k, and so
%    on, until a 0 is reached. Each point i listed in this matrix
%    corresponds to the ith row of vFilamentXYZ, which contains the x/y/z
%    coordinates of the point.
%


% Read vFilamentEdges from desired directory

directory_name = uigetdir('','Select directory containing filament coordinates (exported↙
from Imaris)')
cd(directory_name)
a=csvread('vFilamentXYZ.txt');
c=csvread('vFilamentEdges.txt');
sizea=size(a);
upp=sizea(1);
c2=c;

% Construct the matrix d2:
% -The ith row of d2 corresponds to the ith point defined in vFilamentXYZ.
% -Row i, Col 1 corresponds to how many segments the ith point is part of
% -Row i, Col 2 serves as a counter of how many segments containing the ith
%  point remain. The value of Row i, Col 2 therefore starts off equal to
%  Row i, Col 1; but, as each of the segments is counted, this value will
%  reduce by 1 until eventually it reaches 0.
% -Row i, Col 3 equals 0 if the ith point is not a branch or end point
```

```
% (i.e. it belongs to exactly two segments), and equals 1 otherwise.
% -Row i, Col 4:m lists all the points that the ith point is connected to.
%  Zeros are used as placeholders so that the same number of columns are
%  used in each row.
% -Row i, Col m+j for j>1 serves as a marker as a counter of whether the
%  segment containing the ith point and the point located at Row i, Col 3+j
%  has already been counted. This value starts off equal to 1, but is
%  reduced to 0 after the segment is counted.
for i=1:upp
    [row col]=find(c2==i);
    tester=size(row);
    numtester=tester(1);
    d2(i,1)=numtester;
    for j=1:numtester
        d2(i,3+j)=c2(row(j),3-col(j));
    end
end
d2(:,2)=d2(:,1);
d2(:,3)=(d2(:,1)~=2);
width=max(d2(:,1));
d2(:,(4+width):(3+(2*width)))=(d2(:,4:(3+width))>0);

% Initialize looping parameters
looptest=1;
ptnum=0;
tracknum=1;

while looptest
    % Find branch or end point "currpt" that still belongs to an un-counted
    % segment
    [row col]=find((d2(:,2)).*(d2(:,3)));
    looptest2=1;
    currpt=row(1);
    % Reduce the counter at d2 Row currpt, Col 2 by 1.
    d2(currpt,2)=(d2(currpt,2)-1);
    % Start a new track with point "currpt" as the first point
    tracks(ptnum+1,tracknum)=currpt;
    ptnum=ptnum+1;
    while looptest2
        % Let prevpt = currpt from the last iteration
        prevpt=currpt;
        % Find an uncounted segment containing point "prevpt"
        [row col]=find(d2(prevpt,(4+width):(3+(2*width))));
        % Make this point the new currpt, and mark the points of this
        % segment as counted in both places (i.e. Rows "prevpt" and
        % "currpt")
        d2(prevpt,3+width+col(1))=0;
        currpt=d2(prevpt,3+col(1));
        [row col]=find(d2(currpt,(4:3+width))==prevpt);
        d2(currpt,3+width+col(1))=0;
        d2(currpt,2)=((d2(currpt,2))-1);
        % Add this new point to the current track and iterate ptnum
```

```
        tracks(ptnum+1,tracknum)=currpt;
        ptnum=ptnum+1;
        % Only continue this track if currpt is not a branch or end point
        looptest2=d2(currpt,1)==2;
    end
    % Only continue making tracks if there are still uncounted segments
    looptest=((sum((d2(:,2)).*(d2(:,3))))>0);
    % Reset ptnum, iterate tracknum
    ptnum=0;
    tracknum=tracknum+1;
end

% Eliminate tracks containing only two points
trklength=sum(tracks>0);
[row col]=find(trklength>2);
tracks=tracks(:,col);

% Split tracks that form closed loops into two
trklength=sum(tracks>0);
dummy=size(tracks);
numtracks=dummy(2);
addedtrks=tracks(:,1).*0;
newtracks=0;

for i=1:numtracks
    firstpt=tracks(1,i);
    lastpt=tracks(trklength(i),i);
    if firstpt==lastpt
        midi=floor(trklength(i)/2);
        addedtrks(1:(trklength(i)-midi),newtracks+1)=tracks((midi+1):trklength(i),i);
        newtracks=newtracks+1;
        tracks((midi+1):trklength(i),i)=(tracks((midi+1):trklength(i),i)).*0;
    end
end

if newtracks>0
    tracks(:,numtracks+1:numtracks+newtracks)=addedtrks;
end

trklength=sum(tracks>0);
maxlength=max(trklength);
tracks=tracks(1:maxlength,:);

% Write to file the listing of the tracks

csvwrite('tracks.txt',tracks);

end
```
