# Supplementary material for: A Specialized Microvascular Domain in the Mouse Neural Stem Cell Niche
Source: PLoS One. 2013 Jan 7;8(1):e53546. doi: 10.1371/journal.pone.0053546 (PMC3538546; doi:10.1371/journal.pone.0053546)
Supplement: Script S3 — MATLAB script for calculating vessel tortuosity. What follows is a MATLAB script that takes filament coordinates exported by Imaris 7.4.2 (Bitplane) and the tracks calculated by “calctracks.m”, and calculates vessel tortuosity for each track. Filament coordinates can be exported using ImarisXT. (PDF) [file pone.0053546.s007.pdf]

```
function calctortuosity

% calctortuosity.m
%
% Copyright 2010-2012,
% Baylor College of Medicine
% All Rights Reserved
%
% Last modified 7/9/2012
%
% Takes filament coordinates exported by Imaris 7.4.2 (Bitplane) and the
% tracks calculated by "calctracks.m", and calculates vessel tortuosity for
% each track. Filament coordinates can be exported using ImarisXT.
%
%    Inputs:
%    -Directory containing vessel coordinates and tracks
%    -Directory containing filament coordinates that define reference plane
%     or alternatively the equation of a plane. Used for rotation of data
%     and plotting of the plane.
%
%    Output:
%    "arclength.txt" - csv formatted file listing the arclength for each
%                      track
%    "displac.txt" - csv formatted file listing the total displacement for
%                    each track
%    "tort.txt" - csv formatted file listing the tortuosity for each
%                 track
%    "zcom.txt" - csv formatted file listing the z-center of mass for each
%                 track. This is in rotated coordinates, where z represents
%                 distance from the reference plane.
%


% Read vFilamentXYZ and tracks from desired directory

directory_name = uigetdir('','Select directory containing filament coordinates (exported from Imaris)')
cd(directory_name)
a=csvread('vFilamentXYZ.txt');
tracks=csvread('tracks.txt');

% Decide how to define reference plane
 button3 = questdlg('Define equation of the plane manually, or by finding plane of best fit from saved filament coordinates?','','Manual','Plane of Best Fit','Manual');
    if button3
        fitplanetest=button3(1)=='P';
    end
% If defining the plane using the plane of best fit through saved filament
% coordinates, loads filament coordinates from given directory
    if fitplanetest
        directory_name = uigetdir('','Select directory containing the filaments that define the reference plane (exported from Imaris)')
```

```
        vFilamentXYZ=csvread(strcat(directory_name,'\vFilamentXYZ.txt'));
        Xcolv = vFilamentXYZ(:,1);
        Ycolv = vFilamentXYZ(:,2);
        Zcolv = vFilamentXYZ(:,3);
        Const = ones(size(Xcolv));
% Use regression to determine plane of best fit
        Coefficients = [Xcolv Ycolv Const]\Zcolv;
        XCoeff = Coefficients(1);
        YCoeff = Coefficients(2);
        CCoeff = Coefficients(3);
    else
% If defining the plane manually, queries the coefficients. Default is
% a=b=c=0
        prompt2 = {'Z = aX + bY + c↙
a =','b =','c ='};
        dlg_title2 = 'Define plane coefficients';
        num_lines2 = 1;
        def2 = {'0','0','0'};
        answer2 = inputdlg(prompt,dlg_title,num_lines,def);
        if ~isempty(answer2)
            XCoeff = str2num(cell2mat(answer2(1)));
            YCoeff = str2num(cell2mat(answer2(2)));
            CCoeff = str2num(cell2mat(answer2(3)));
        end
    end

vFilamentXYZ=a;

% Now, rotate the data with respect to the reference plane...
% We start by defining the normal vector to the plane.
vector=[-XCoeff -YCoeff 1];
arclength=sqrt(dot(vector,vector));

% Next, we determine what angle this vector must be rotated by in order for
% it to lie in the x-y plane. After determining this angle (-phi), we
% rotate the vector by -phi around the x-axis by multiplying by the
% appropriate rotation matrix.
phi=atan2(vector(1,3),vector(1,2));
vector2=[1,0,0;0,cos(-phi),(-sin(-phi));0,sin(-phi),cos(-phi)]*vector';
vector2=vector2';

% Then, we determine what angle this transformed vector must be rotated by
% in order for it to lie parallel with the x-axis. After determining this
% angle (-theta), we are ready to rotate the coordinates of the vessels.
theta=atan2(vector2(1,2),vector2(1,1));

% Now, we rotate the coordinates of the vessels. We start by rotating
% around the x-axis by -phi.
vFilamentXYZ=[1,0,0;0,cos(-phi),(-sin(-phi));0,sin(-phi),cos(-phi)]*vFilamentXYZ';

% Next, we rotate around the z-axis by -theta.
vFilamentXYZ=[cos(-theta),(-sin(-theta)),0;sin(-theta),cos(-theta),0;0,0,1]*vFilamentXYZ;
```

```
% Finally, we rotate around the y-axis by -90 degrees. This is required so
% that the vector normal to the plane is parallel with the z-axis instead
% of the x-axis. This is a special case of the corresponding rotation
% matrix.
vFilamentXYZ=vFilamentXYZ';
xb=vFilamentXYZ(:,1);
yb=vFilamentXYZ(:,2);
zb=vFilamentXYZ(:,3);
vFilamentXYZ(:,3)=xb;
vFilamentXYZ(:,1)=-zb;

% To zero our transformed coordinates such that the reference plane is
% located at z=0, we determine at what z-location the plane sits after the
% rotations above. Because z is constant after the rotations, all we need
% to do is calculate z for any point on the plane. We do this with the
% point (x,y,z)=(0,0,c).
PlaneCoord=CCoeff*sin(phi)*sin(theta);

% Then, we subtract the z-values of our rotated data set by this constant.
vFilamentXYZ(:,3)=vFilamentXYZ(:,3)-PlaneCoord;

% Now we use tracks to calculate tortuosity for each vessel segment. We
% also save the distance between the reference plane and the center of mass
% of this segment

a=vFilamentXYZ;
a2=a;

clear arclength

% Determine the number of tracks, and the length of each track
trklength=sum(tracks>0);
testsize=size(trklength);
tracknum=testsize(2);

% Initialize arrays
arclength=trklength.*0;
displac=arclength;
tort=arclength;
zcom=arclength;

for i=1:tracknum
    ds=0;
    % Start a running sum of the z-coordinates
    zcom(1,i)=zcom(1,i)+a2(tracks(1,i),3);
    for j=1:(trklength(1,i)-1)
        % Determine the endpoints of the current segment
        x2=a2(tracks(j+1,i),1);
        y2=a2(tracks(j+1,i),2);
        z2=a2(tracks(j+1,i),3);
        x1=a2(tracks(j,i),1);
```

```
        y1=a2(tracks(j,i),2);
        z1=a2(tracks(j,i),3);
        % Determine the length of the current segment
        dx=x2-x1;
        dy=y2-y1;
        dz=z2-z1;
        ds0=sqrt((dx^2)+(dy^2)+(dz^2));
        % Add this to a running total of arclength
        ds=ds+ds0;
        % Continue the running sum of the z-coordinate
        zcom(1,i)=zcom(1,i)+z2;
    end
    % Store arclength for this track
    arclength(1,i)=ds;
    % Divide z-coord running sum by # of points to get z-center of mass.
    zcom(1,i)=zcom(1,i)/(trklength(1,i)-1);
    % Determine the first and last points of the entire track
    x2=a2(tracks(trklength(1,i),i),1);
    y2=a2(tracks(trklength(1,i),i),2);
    z2=a2(tracks(trklength(1,i),i),3);
    x1=a2(tracks(1,i),1);
    y1=a2(tracks(1,i),2);
    z1=a2(tracks(1,i),3);
    % Determine the total displacement between these two points
    dx=x2-x1;
    dy=y2-y1;
    dz=z2-z1;
    dsdisp=sqrt((dx^2)+(dy^2)+(dz^2));
    % Store total displacement for this track
    displac(1,i)=dsdisp;
    % Calculate and store tortuosity for this track (arclength/displacemt)
    tort(1,i)=arclength(1,i)/displac(1,i);
end

% Save calculated parameters to file
csvwrite('arclength.txt',arclength);
csvwrite('displac.txt',displac);
csvwrite('tort.txt',tort);
csvwrite('zcom.txt',zcom);

AvgTortuosity=mean(tort)

end
```
