# Supplementary material for: A Specialized Microvascular Domain in the Mouse Neural Stem Cell Niche
Source: PLoS One. 2013 Jan 7;8(1):e53546. doi: 10.1371/journal.pone.0053546 (PMC3538546; doi:10.1371/journal.pone.0053546)
Supplement: Script S4 — MATLAB script for plotting vessel networks and calculating vessel density or angle with respect to a reference plane. What follows is a MATLAB script that plots a vessel network using exported filament coordinates from Imaris 7.4.2 (Bitplane) and the tracks calculated by “calctracks.m”. Filament coordinates can be exported using ImarisXT. The script can then use the plotted vessel network to calculate vessel density or angle with respect to a reference plane. (PDF) [file pone.0053546.s008.pdf]

```
function output = vesselrender

% vesselrender.m
%
% Copyright 2010-2012,
% Baylor College of Medicine
% All Rights Reserved
%
% Last modified 7/8/2012
%
% Renders a vessel network in MATLAB R2008a (MathWorks) using exported
% filament coordinates from Imaris 7.4.2 (Bitplane) and the
% tracks calculated by "calctracks.m". Filament coordinates can be
% exported using ImarisXT.
%
% Program can run in three modes (selected using dialog box):
% A) Plot Vessels - Plots a 3D vessel network in MATLAB
%                   Also gives the option to plot a reference plane
%    Inputs:
%    -Directory containing vessel coordinates
%    -Directory containing filament coordinates that define reference plane
%     or alternatively the equation of a plane. Used for rotation of data
%     and plotting of the plane. Only needed if plotting plane.
%
%    Outputs:
%    -3D plot of vessels and plane
%
% B) Analyze Density - Calculates vessel density as a function of
%                      perpendicular distance from a reference plane
%    Inputs:
%    -Directory containing vessel coordinates
%    -Directory containing filament coordinates that define reference plane
%     or alternatively the equation of a plane
%    -Directory for saving output files
%    -xmin/xmax/ymin/ymax/zmin/zmax for the volume to be analyzed
%    -Sampling frequency (axial distance between adjacent cross-sections)
%
%    Outputs:
%     "FigXXX.fig" - MATLAB figures displaying cross-sectional planes
%       through the plotted 3D volume
%     "VesselArea.txt" - csv formatted file containing vessel cross-
%       sectional areas as a function of distance from reference plane
%     "TotalArea.txt" - csv formatted file containing cross-sectional areas
%       of total volume as a function of distance from reference plane
%     "AllAreaData.txt" - csv formatted file containing cross-sectional
%       areas of the vessels and total volume, as well as their ratio,
%       as a function of distance from reference plane
%     "DensityProfile.fig" - MATLAB figure plotting the vessel volume
%       density as a function of distance from reference plane
%
% C) Analyze Angle - Calculates angle between vessel tracks and a reference
%                    plane
```

```
%    Inputs:
%    -Directory containing vessel coordinates
%    -Directory containing filament coordinates that define reference plane
%     or alternatively the equation of a plane
%    -Directory for saving output files
%
%    Outputs:
%     "AngleProfile.fig" - MATLAB figure displaying a scatterplot of
%       angles from reference plane as a function of distance from plane
%     "AngleData.txt" - csv formatted file containing angles from reference
%       plane as a function of distance from plane
%     "CumAngle.fig" - MATLAB figure displaying the cumulative probability
%       distribution of measured angles
%     "CumAngle.txt" - csv formatted file containing the cumulative
%       probability distribution of measured angles
%

%%%%%%%%%%%%%%%%%%%%%%%%%%%%%%%%%%%%%%%%%%%%%%%%%%%%%%%
% Part 1 - Select run-mode and define reference plane
%
% Start by setting the defaults
modetest=0;
analysismode=0;
planetest=1;
fitplanetest=0;

% Determine whether to plot vessels, analyze density, or analyze planarity.
% Default is to plot vessels.
button = questdlg('What do you want to do with vessel coordinates?','Select Mode','Plot↙
Vessels','Analyze Density','Analyze Planarity','Plot Vessels');
if button
    modetest=button(1)=='A';
end
if modetest
    analysismode=button(9)=='P';
else
% If plotting vessels, decide whether to plot reference plane. Default is yes.
    button2 = questdlg('Plot reference plane?','','Yes','No','Yes');
    if button2
        planetest=button2(1)=='Y';
    end
end

% Define equation for reference plane. Reference plane is defined, even if
% not being plotted, because it is necessary for rotation of the volume,
% and potentially for analysis of density as a distance from the plane, or
% for determination for path angle with respect to the plane. Coefficients
% are for the following equation of the plane: Z = aX + bY + c
% The vector orthogonal to the plane must have a non-zero z-component.


% Set default plane to be Z = 0. When using this default, the data set will
```

```
% not be rotated at all.

XCoeff = 0; % a = X coefficient
YCoeff = 0; % b = Y coefficient
CCoeff = 0; % c = constant term

% If plotting the plane, analyzing density, or analyzing angle, discards
% default plane and loads user-defined plane.

if planetest
% Determines whether to define plane manually, or by finding plane of
% best-fit through saved filament coordinates. Default is manual.
    button3 = questdlg('Define equation of the plane manually, or by finding plane of best ↙
fit from saved filament coordinates?','','Manual','Plane of Best Fit','Manual');
    if button3
        fitplanetest=button3(1)=='P';
    end
% If defining the plane using the plane of best fit through saved filament
% coordinates, loads filament coordinates from given directory
    if fitplanetest
        directory_name = uigetdir('','Select directory containing the filaments that↙
define the reference plane (exported from Imaris)')
        vFilamentXYZ=csvread(strcat(directory_name,'\vFilamentXYZ.txt'));
        Xcolv = vFilamentXYZ(:,1);
        Ycolv = vFilamentXYZ(:,2);
        Zcolv = vFilamentXYZ(:,3);
        Const = ones(size(Xcolv));
% Use regression to determine plane of best fit
        Coefficients = [Xcolv Ycolv Const]\Zcolv;
        XCoeff = Coefficients(1);
        YCoeff = Coefficients(2);
        CCoeff = Coefficients(3);
    else
% If defining the plane manually, queries the coefficients. Default is
% a=b=c=0
        prompt2 = {'Z = aX + bY + c↙
a =','b =','c ='};
        dlg_title2 = 'Define plane coefficients';
        num_lines2 = 1;
        def2 = {'0','0','0'};
        answer2 = inputdlg(prompt,dlg_title,num_lines,def);
        if ~isempty(answer2)
            XCoeff = str2num(cell2mat(answer2(1)));
            YCoeff = str2num(cell2mat(answer2(2)));
            CCoeff = str2num(cell2mat(answer2(3)));
        end
    end

end

% Gets directory containing the coordinates of the vessel network. This
% information should be exported using ImarisXT and should include
```

```
% vFilamentXYZ.txt, tracks.txt, and vFilamentRadius.txt
directory_name2 = uigetdir('','Select directory containing the filaments that define the↙
vessels (exported from Imaris)');

vFilamentXYZ=csvread(strcat(directory_name2,'\vFilamentXYZ.txt'));
tracks=csvread(strcat(directory_name2,'\tracks.txt'));
vFilamentRadius=csvread(strcat(directory_name2,'\vFilamentRadius.txt'));

% Guesses the x-, y-, and z- limits for the volume that is loaded. This is
% important for determining vessel density with respect to reference plane,
% because after rotation, the total area of each slice will not be equal at
% different distances from the reference plane. Also sets the default slice
% interval to be 1 um.
xmax=max(vFilamentXYZ(:,1));
xmin=min(vFilamentXYZ(:,1));
ymax=max(vFilamentXYZ(:,2));
ymin=min(vFilamentXYZ(:,2));
zmax=max(vFilamentXYZ(:,3));
zmin=min(vFilamentXYZ(:,3));
sliceint=1;

if modetest
% Sets the directory for saving files - only used when analyzing density or angle
    savedir = uigetdir('','Select directory where files will be saved');
    if savedir==0
        'Error - Must select directory where files will be saved'
        return
    end
% Queries the x-, y-, and z- limits for the volume that is loaded. The
% default values will only be close if the entire volume is filled with
% vessels, so use them with care. Sampling interval is also important - a
% smaller value will improve resolution, but requires more memory and
% runtime.
% This step is only necessary if in density analysis mode.
    if ~analysismode
        prompt = {'Xmin (um)','Xmax (um)','Ymin (um)','Ymax (um)','Zmin (um)','Zmax↙
(um)','Sampling Interval (um)'};
        dlg_title = 'Data Limits';
        num_lines = 1;
        def = {'0',num2str(xmax),'0',num2str(ymax),'0',num2str(zmax),'1'};
        answer = inputdlg(prompt,dlg_title,num_lines,def);
        if ~isempty(answer)
            xmin=str2num(cell2mat(answer(1)));
            xmax=str2num(cell2mat(answer(2)));
            ymin=str2num(cell2mat(answer(3)));
            ymax=str2num(cell2mat(answer(4)));
            zmin=str2num(cell2mat(answer(5)));
            zmax=str2num(cell2mat(answer(6)));
            sliceint=str2num(cell2mat(answer(7)));
        end
    end
end
```

```
%%%%%%%%%%%%%%%%%%%%%%%%%%%%%%%%%%%%%%%%%%%%%%%%%%%%%%%
% Part 2 - Rotate and translate entire data set so that the reference plane
% is parallel with the plane Z = 0. This transforms the z-coordinates so
% that z= perpendicular distance from the reference plane.
%
% To do this, we must first determine what rotations are necessary to
% rotate the normal vector to the reference plane so that it transforms to
% [0 0 1]. Then we must rotate all of the vessel coordinates by these same
% angles. Because 3D rotations are NOT commutative, order is important
% here. Finally, the rotated coordinates must be shifted in z so that the
% transformed reference plane is located at z=0.
%

% We start by defining the normal vector to the plane.
vector=[-XCoeff -YCoeff 1];
arclength=sqrt(dot(vector,vector));

% Next, we determine what angle this vector must be rotated by in order for
% it to lie in the x-y plane. After determining this angle (-phi), we
% rotate the vector by -phi around the x-axis by multiplying by the
% appropriate rotation matrix.
phi=atan2(vector(1,3),vector(1,2));
vector2=[1,0,0;0,cos(-phi),(-sin(-phi));0,sin(-phi),cos(-phi)]*vector';
vector2=vector2';

% Then, we determine what angle this transformed vector must be rotated by
% in order for it to lie parallel with the x-axis. After determining this
% angle (-theta), we are ready to rotate the coordinates of the vessels.
theta=atan2(vector2(1,2),vector2(1,1));

% Now, we rotate the coordinates of the vessels. We start by rotating
% around the x-axis by -phi.
vFilamentXYZ=[1,0,0;0,cos(-phi),(-sin(-phi));0,sin(-phi),cos(-phi)]*vFilamentXYZ';

% Next, we rotate around the z-axis by -theta.
vFilamentXYZ=[cos(-theta),(-sin(-theta)),0;sin(-theta),cos(-theta),0;0,0,1]*vFilamentXYZ;

% Finally, we rotate around the y-axis by -90 degrees. This is required so
% that the vector normal to the plane is parallel with the z-axis instead
% of the x-axis. This is a special case of the corresponding rotation
% matrix.
vFilamentXYZ=vFilamentXYZ';
xb=vFilamentXYZ(:,1);
yb=vFilamentXYZ(:,2);
zb=vFilamentXYZ(:,3);
vFilamentXYZ(:,3)=xb;
vFilamentXYZ(:,1)=-zb;

% To zero our transformed coordinates such that the reference plane is
% located at z=0, we determine at what z-location the plane sits after the
% rotations above. Because z is constant after the rotations, all we need
```

```
% to do is calculate z for any point on the plane. We do this with the
% point (x,y,z)=(0,0,c).
PlaneCoord=CCoeff*sin(phi)*sin(theta);

% Then, we subtract the z-values of our rotated data set by this constant.
vFilamentXYZ(:,3)=vFilamentXYZ(:,3)-PlaneCoord;

% In order to calculate density with respect to the distance from the
% reference plane, a surface that bounds the volume must be defined, and
% then these same transformations must be applied.
%
% The surface is defined by the vertices of the rectangular prism bounding
% the data set, and by the centroids of the upper and lower faces of this
% rectangular prism. These faces are required in order to close the
% surface, so that contours can be drawn.
x=[xmin xmax xmax xmin];
y=[ymin ymin ymax ymax];
x=[x;x];
y=[y;y];
z=[zmin zmin zmin zmin;zmax zmax zmax zmax];
z=[z(1,:);z;z(2,:)];
xdel=(xmin+((xmax-xmin)/2));
ydel=(ymin+((ymax-ymin)/2));
x=[xdel xdel xdel xdel;x;xdel xdel xdel xdel];
y=[ydel ydel ydel ydel;y;ydel ydel ydel ydel];
x(:,5)=x(:,1);
y(:,5)=y(:,1);
z(:,5)=z(:,1);

coord1=[x(1,:);y(1,:);z(1,:)];
coord2=[x(2,:);y(2,:);z(2,:)];
coord3=[x(3,:);y(3,:);z(3,:)];
coord4=[x(4,:);y(4,:);z(4,:)];

% Then, the same transformations are applied to the coordinates defining
% this surface.
coord1t=[1,0,0;0,cos(-phi),(-sin(-phi));0,sin(-phi),cos(-phi)]*coord1;
coord2t=[1,0,0;0,cos(-phi),(-sin(-phi));0,sin(-phi),cos(-phi)]*coord2;
coord3t=[1,0,0;0,cos(-phi),(-sin(-phi));0,sin(-phi),cos(-phi)]*coord3;
coord4t=[1,0,0;0,cos(-phi),(-sin(-phi));0,sin(-phi),cos(-phi)]*coord4;
coord1tt=[cos(-theta),(-sin(-theta)),0;sin(-theta),cos(-theta),0;0,0,1]*coord1t;
coord2tt=[cos(-theta),(-sin(-theta)),0;sin(-theta),cos(-theta),0;0,0,1]*coord2t;
coord3tt=[cos(-theta),(-sin(-theta)),0;sin(-theta),cos(-theta),0;0,0,1]*coord3t;
coord4tt=[cos(-theta),(-sin(-theta)),0;sin(-theta),cos(-theta),0;0,0,1]*coord4t;

xdiamond=[coord1tt(1,:);coord2tt(1,:);coord3tt(1,:);coord4tt(1,:);];
ydiamond=[coord1tt(2,:);coord2tt(2,:);coord3tt(2,:);coord4tt(2,:);];
zdiamond=[coord1tt(3,:);coord2tt(3,:);coord3tt(3,:);coord4tt(3,:);];

xdiamondb=xdiamond;
ydiamondb=ydiamond;
zdiamondb=zdiamond;
```

```
zdiamond=xdiamondb;
xdiamond=-zdiamondb;

zdiamond=zdiamond-PlaneCoord;

% Finally, determine the x-, y-, and z-limits, and use this and the slice
% interval to determine the number of slices required
xmax=max(max(xdiamond));
xmin=min(min(xdiamond));
ymax=max(max(ydiamond));
ymin=min(min(ydiamond));
zmax=max(max(zdiamond));
zmin=min(min(zdiamond));

zdelta=zmax-zmin;

numslices=floor(zdelta/sliceint);

v=zeros(1,numslices);

for i=1:numslices
    v(1,i)=i;
end

v=v-1;
v=v.*sliceint*(-1);
v=v+zmax;
v=rot90(v);

%%%%%%%%%%%%%%%%%%%%%%%%%%%%%%%%%%%%%%%%%%%%%%%%%%%%%%%
% Part 3 - Plot the entire vessel network, and use it to either display the
% vessel network, analyze the density, or analyze the angle (as selected in
% Part 1 above).

% Use the list of tracks to determine how many tracks there are, and how
% many points in each track.
trklength=sum(tracks>0);
testsize=size(trklength);
tracknum=testsize(2);

% Open a figure, and set defaults.
figure;
[x y z]=cylinder([1 2]);
xold=x(1,:).*0;
yold=xold;
zold=xold;
if analysismode
    planarity=[];
end

% Draw each track segment by segment. i=track number, and j=point number
% within the current track
```

```
for i=1:tracknum
    for j=1:(trklength(1,i)-1)
% Determine the location of points j and j+1 along this track, as well as
% the radius of the filament at each of these points. Define a vector using
% these two points.
        xyz2=[vFilamentXYZ(tracks(j+1,i),1),vFilamentXYZ(tracks(j+1,i),2),vFilamentXYZ ↙
(tracks(j+1,i),3)];
        xyz1=[vFilamentXYZ(tracks(j,i),1),vFilamentXYZ(tracks(j,i),2),vFilamentXYZ(tracks ↙
(j,i),3)];
        r2=vFilamentRadius(tracks(j+1,i),1);
        r1=vFilamentRadius(tracks(j,i),1);
        vector=xyz2-xyz1;
        arclength=norm(vector);
% If run-mode is for determining angle, use the dot product of this vector
% with the dot product of the vector normal to the reference plane [0 0 1]
% to determine the angle. Add this to the list and go to the next track
% point.
        if analysismode
            wallangle=((acos(vector(1,3)/arclength))*180/pi)-90;
            planarity=[planarity;((xyz1(1,3)+xyz2(1,3))/2),abs(wallangle)];
            continue
        end

% To plot each vessel segment, we will use the MATLAB cylinder function,
% which plots a cylinder with an axis of (x,y,z) = (0,0,0) to (0,0,1), and
% a user-defined radius at each end of the axis. This cylinder then needs
% to be rotated +90 degrees around the y-axis, and then rotated again by an
% angle of theta around the z-axis, and then one last time by an angle of
% phi around the x-axis. Finally, it will have to be translated from the
% origin to the appropriate location in 3-space.
%
% To determine phi and theta, we perform the inverse by determining what
% angle the vector of the segment must be rotated by in order to move it to
% the x-y plane, and then from there to be parallel with the x-axis.
% Remember that 3D rotation is not commutative, so these rotations must be
% done in reverse when moving the cylinder from the z-axis to its
% appropriate angle and location with respect to the origin.
        phi=atan2(vector(1,3),vector(1,2));
        vector2=[1,0,0;0,cos(-phi),(-sin(-phi));0,sin(-phi),cos(-phi)]*vector';
        vector2=vector2';
        theta=atan2(vector2(1,2),vector2(1,1));

% Here, we use the cylinder function to determine the coordinates of our
% cylinder with the appropriate length and radii, albeit at the wrong angle
% and location.
        [x y z]=cylinder([r1 r2]);
        z(2,:)=arclength;

% If this is not the first segment in this track, then it must be axially
% rotated so that it will be axially aligned with the previous segment.
% Otherwise, the internal volume of the cylindrical segment will be
% artificially reduced because the walls will be twisted together.
```

```
% For example, from    -----------    to    ----  ----
%                                                \/
%                                                /\
%                      -----------          ----  ----
%
% Determine the angle psi that the new cylinder must be axially rotated by,
% and then perform the rotation.
        if j>1
            testvec=[(xold(1,1)-xyz1(1,1)),(yold(1,1)-xyz1(1,2)),(zold(1,1)-xyz1(1,3))];
            testvecA=[1,0,0;0,cos(-phi),(-sin(-phi));0,sin(-phi),cos(-phi)]*testvec';
            testvecB=[cos(-theta),(-sin(-theta)),0;sin(-theta),cos(-theta),0;0,0,1] ↙
*testvecA;
            testvecC=[-testvecB(3,1);testvecB(2,1);testvecB(1,1)];
            psi=atan2(testvecC(2,1),testvecC(1,1));
            coord1=[x(1,:);y(1,:);z(1,:)];
            coord2=[x(2,:);y(2,:);z(2,:)];
            coord1tt=[cos(psi),(-sin(psi)),0;sin(psi),cos(psi),0;0,0,1]*coord1;
            coord2tt=[cos(psi),(-sin(psi)),0;sin(psi),cos(psi),0;0,0,1]*coord2;
            xtt=[coord1tt(1,:);coord2tt(1,:)];
            ytt=[coord1tt(2,:);coord2tt(2,:)];
            ztt=[coord1tt(3,:);coord2tt(3,:)];
            x=xtt;y=ytt;z=ztt;
        end

% Now, we use the angles we determined above to transform the coordinates
% of our cylinder so that it is rotated at the appropriate angle.
%
% We start by rotating by +90 degrees around the y-axis.
        xb=x;
        yb=y;
        zb=z;
        z=-xb;
        x=zb;
        coord1=[x(1,:);y(1,:);z(1,:)];
        coord2=[x(2,:);y(2,:);z(2,:)];
% Next, we rotate by theta around the z-axis
        coord1t=[cos(theta),(-sin(theta)),0;sin(theta),cos(theta),0;0,0,1]*coord1;
        coord2t=[cos(theta),(-sin(theta)),0;sin(theta),cos(theta),0;0,0,1]*coord2;
% Then, we rotate by phi around the x-axis
        coord1tt=[1,0,0;0,cos(phi),(-sin(phi));0,sin(phi),cos(phi)]*coord1t;
        coord2tt=[1,0,0;0,cos(phi),(-sin(phi));0,sin(phi),cos(phi)]*coord2t;
        xtt=[coord1tt(1,:);coord2tt(1,:)];
        ytt=[coord1tt(2,:);coord2tt(2,:)];
        ztt=[coord1tt(3,:);coord2tt(3,:)];
% Finally, we translate the cylinder so that it is located at the right
% location in 3-space.
        xfinal=xtt+xyz1(1,1);
        yfinal=ytt+xyz1(1,2);
        zfinal=ztt+xyz1(1,3);
% If this is the second (or later) segment within this track, we must
% replace the coordinates of the beginning edge of the cylinder with the
% coordinates used previously for the ending edge of the cylinder. This
```

```
% ensures continuity of vessel filament from segment to segment, with no
% gap in between segments.
        if j>1
            xfinal(1,:)=xold;
            yfinal(1,:)=yold;
            zfinal(1,:)=zold;
        end

% The coordinates for both faces of the cylinder must be added to the list
% of cylinder coordinates. This ensures a closed surface, and makes sure that
% contours can be drawn.
        xfinal=[((xfinal(1,:).*0)+xyz1(1,1));xfinal;((xfinal(1,:).*0)+xyz2(1,1))];
        yfinal=[((yfinal(1,:).*0)+xyz1(1,2));yfinal;((yfinal(1,:).*0)+xyz2(1,2))];
        zfinal=[((zfinal(1,:).*0)+xyz1(1,3));zfinal;((zfinal(1,:).*0)+xyz2(1,3))];
% The coordinates for the ending edge of the cylinder are saved, so that
% they can be used as the beginning coordinates of the cylinder in the next
% iteration.
        xold=xfinal(3,:);
        yold=yfinal(3,:);
        zold=zfinal(3,:);

% If run-mode is for displaying a plot of the vessels, use the surf
% function to draw this filament segment, and then go on to the next segment.
        if ~modetest
            surf(xfinal,yfinal,zfinal,'FaceColor',[1 0 0],'EdgeColor',[0 0 0]);
            hold on
            continue
        end

% Otherwise, run-mode must be set for analyzing density. Use the contour3
% mode instead in order to draw contours, which can be used for determining
% cross-sectional area of the vessel network at each z-plane of interest,
% as was defined above.
        [c h]=contour3(xfinal,yfinal,zfinal,v,'k');
        hold on
% Each of these contours is saved as a graphics object with handle h. For
% each of these unique handles, save tags marking these contours as
% belonging to a vessel, and with tags that note the z-coordinate where the
% contour was taken. These tags will facilitate analysis of density as a
% function of z (which after our transformations, represents distance from
% the z-plane).
        sizeh1=size(h);
        sizeh2=sizeh1(1,1);
        for iii=1:sizeh2
            a=get(h(iii),'ZData');
            height=a(1,1);
            set(h(iii),'UserData',height);
            set(h(iii),'Tag','Vessel');
        end
    end
% Track progress as vessel tracks are plotted.
VesselPlotProgress=i/tracknum
```

```
end

% If run-mode is for displaying a plot of the vessel network, then plot the
% reference plane if desired, and then end program.
if ~modetest
    if planetest
        [xxp, yyp]=meshgrid(xmin:((xmax-xmin)/10):xmax,ymin:((ymax-ymin)/10):ymax);
        zzp = 0 * xxp;
        surf(xxp,yyp,zzp,'FaceColor',[0 0 1],'EdgeColor',[0 0 0])
        hold on
    end
    daspect([1 1 1]);
    output='Finished Plotting Vessels'
    return
end

% If run-mode is for analyzing angle, plot a scatterplot of vessel angle
% with respect to distance from reference plane. Then, plot the cumulative
% probability distribution of vessel angle. Finally, save these figures,
% and the data that make up these tables as comma separated value text
% files, and end program.
if analysismode
    planarity(:,1)=planarity(:,1);
    xlim([min(planarity(:,1))-5 max(planarity(:,1))+5]);ylim([-5 95]);plot(planarity(:,1), ↙
planarity(:,2),'kd');hold on;
    title('Vessel Angle from Plane as a Function of Depth');
    xlabel('Depth (um)');
    ylabel('Angle (degrees)');
    hgsave(strcat(savedir,'\AngleProfile.fig'));
    csvwrite(strcat(savedir,'\AngleData.txt'),planarity);

    cumangle=[10 20 30 40 50 60 70 80 90]';
    plnsize=size(planarity);
    plnsize2=plnsize(1,1);
    for i=1:9
        cumangle(i,2)=sum(planarity(:,2)<cumangle(i,1));
    end
    cumangle(:,2)=cumangle(:,2)/plnsize2;
    cumangle=[0 0;cumangle];
    figure;plot(cumangle(:,1),cumangle(:,2));
    title('Cumulative Probability Distribution of Vessel Angle');
    xlabel('Angle (degrees)');
    ylabel('Cumulative Probability');
    hgsave(strcat(savedir,'\CumAngle.fig'));
    csvwrite(strcat(savedir,'\CumAngle.txt'),cumangle);
    output='Finished Angle Measurements'
    return
end

% If program has gotten to this line, then run-mode must be for analyzing
% density. Countours must now be drawn for the surface that bounds the
% volume.
```

```
[c h]=contour3(xdiamond,ydiamond,zdiamond,v,'b');
hold on
% Again, each contour is saved as a graphics object with handle h. For
% each of these unique handles, save tags marking these contours as
% belonging to the frame, and with tags that note the z-coordinate where
% contour was taken. These tags will facilitate analysis of density as a
% function of z (which after our transformations, represents distance from
% the z-plane).
sizeh1=size(h);
sizeh2=sizeh1(1,1);
for iii=1:sizeh2
    a=get(h(iii),'ZData');
    height=a(1,1);
    set(h(iii),'UserData',height);
    set(h(iii),'Tag','Frame');
end

% Make sure data aspect ratio is correct.
set(gca,'DataAspectRatio',[1 1 1]);

% Find every unique value of 'UserData' -- this should be the list of
% z-locations chosen for measurements of vessel density, as defined earlier
% with slice interval. These values are saved into the first column of areasav.
h=findobj;
a=get(h,'UserData');
a=cell2mat(a);
a=unique(a);
sizea1=size(a);
sizea2=sizea1(1,1);
areasav=a;
areasav(:,2)=0.*a;

% Measure the cross-sectional area of the frame and of the vessel network
% at each z-location
for ii=1:sizea2

% Zero the running-area counts, and open a new figure.
    cumarea=0;
    framearea=0;
    figure;hold on;

% Find all the contour objects for the frame at the current z-location. Use
% the x- and y- coordinates of these contours to determine cross-sectional
% area of the frame at this z-location.
    b=findobj('UserData',a(ii),'-and','Tag','Frame');
    sizeb1=size(b);
    sizeb2=sizeb1(1,1);
    for jj=1:sizeb2
        xtest=get(b(jj),'XData');
        ytest=get(b(jj),'YData');
        index=find(~(isnan(xtest)));
        xtest=xtest(index);
```

```
        ytest=ytest(index);
        framearea=framearea+polyarea(xtest,ytest);
% Plot cross-section of the frame in blue.
        fill(xtest,ytest,'b');
        hold on
    end

% Find all the contour objects for the vessels at the current z-location. Use
% the x- and y- coordinates of these contours to determine cross-sectional
% area of the vessel network at this z-location.
    b=findobj('UserData',a(ii),'-and','Tag','Vessel');
    sizeb1=size(b);
    sizeb2=sizeb1(1,1);
    for jj=1:sizeb2
        xtest=get(b(jj),'XData');
        ytest=get(b(jj),'YData');
        index=find(~(isnan(xtest)));
        xtest=xtest(index);
        ytest=ytest(index);
% Plot cross-section of the vessel network in black.
        fill(xtest,ytest,'k');
        hold on
        cumarea=cumarea+polyarea(xtest,ytest);
    end

% Save an image of the cross-section of the frame and vessels at this
% z-location to enable verification of correct sectioning.
    axis([xmin xmax ymin ymax]);
    hgsave(strcat(savedir,'\Fig',int2str(ii)));
    close;

% Save the cross-sectional area of the frame and vessel network at this
% location into areasav.
    areasav(ii,2)=cumarea;
    areasav(ii,3)=framearea;

% Display the progress of the density calculation process.
    DensCalcProgress=ii/sizea2
end

% Save measurements of vessel area and frame area as a function of distance
% from the reference plane in comma separated value formatted text files.
areasav(:,1)=areasav(:,1);
csvwrite(strcat(savedir,'\VesselArea.txt'),areasav(:,1:2));
csvwrite(strcat(savedir,'\TotalArea.txt'),[areasav(:,1),areasav(:,3)]);
close

% Calculate the ratio of the cross-sectional area of the vessel network to
% the cross-sectional area of the frame at each z-location. When small
% slice interval is used, this is a good estimate of the fraction of space
% taken up by the vessel network (in other words, the vessel density) as a
% function of distance from the reference plane. This is saved into
```

```
% areasavfin as both a fraction and a percentage, and the entire table is
% saved as a comma separated value formatted text file.
index=find(areasav(:,3)>0);
areasavfin=areasav(index,:);
areasavfin(:,4)=areasavfin(:,2)./areasavfin(:,3);
areasavfin(:,5)=areasavfin(:,4)*100;
csvwrite(strcat(savedir,'\AllAreaData.txt'),areasavfin);

% Finally, this vessel density as a function of depth is plotted as a line
% graph, and saved to file.
figure;plot(areasavfin(:,1),areasavfin(:,5));
title('Vessel Density as a Function of Depth');
xlabel('Depth (um)');
ylabel('Vessel Density % (v/v)');
hgsave(strcat(savedir,'\DensityProfile.fig'));

% With that, the program is complete.
output='Finished Density Measurements'
```
